# Reorganization of Respiratory Descending Pathways following Cervical Spinal Partial Section Investigated by Transcranial Magnetic Stimulation in the Rat

**DOI:** 10.1371/journal.pone.0148180

**Published:** 2016-02-01

**Authors:** Stéphane Vinit, Emilie Keomani, Therese B. Deramaudt, Marcel Bonay, Michel Petitjean

**Affiliations:** 1 Université de Versailles Saint-Quentin-en-Yvelines, UFR des Sciences de la Santé – Simone Veil, Montigny-le-Bretonneux, France; 2 Inserm U1179, End:icap, Laboratoire de Physiologie TITAN, Montigny-le-Bretonneux, France; 3 LIA-BAHN (Laboratoire International Associé – Biologie Appliquée Handicap Neuromusculaire), CSM (Centre Scientifique de Monaco), 8 Quai Antoine Ier - 98000, Monaco; 4 Service de Physiologie-Explorations Fonctionnelles; Hôpital Ambroise Paré, Assistance Publique-Hôpitaux de Paris (AP-HP), Groupe Hospitalier Paris Ile-de-France Ouest, Boulogne-Billancourt, France; University of Szeged, HUNGARY

## Abstract

High cervical spinal cord injuries lead to permanent respiratory deficits. One preclinical model of respiratory insufficiency in adult rats is the C2 partial injury which causes unilateral diaphragm paralysis. This model allows the investigation of a particular population of respiratory bulbospinal axons which cross the midline at C3-C6 spinal segment, namely the crossed phrenic pathway. Transcranial magnetic stimulation (TMS) is a non-invasive technique that can be used to study supraspinal descending respiratory pathways in the rat. Interestingly, a lateral C2 injury does not affect the amplitude and latency of the largest motor-evoked potential recorded from the diaphragm (MEPdia) ipsilateral to the injury in response to a single TMS pulse, compared to a sham animal. Although the rhythmic respiratory activity on the contralateral diaphragm is preserved at 7 days post-injury, no diaphragm activity can be recorded on the injured side. However, a profound reorganization of the MEPdia evoked by TMS can be observed. The MEPdia is reduced on the non-injured rather than the injured side. This suggests an increase in ipsilateral phrenic motoneurons excitability. Moreover, correlations between MEPdia amplitude and spontaneous contralateral diaphragmatic activity were observed. The larger diaphragm activity correlated with a larger MEPdia on the injured side, and a smaller MEPdia on the non-injured side. This suggests, for the first time, the occurrence of a functional neuroplasticity process involving changes in motoneuron excitability balance between the injured and non-injured sides at a short post-lesional delay.

## Introduction

High cervical spinal cord injuries often lead to respiratory insufficiencies, and ventilatory assistance is necessary in order for the patients to survive [1]. However, few patients can be weaned off the ventilatory assistance, demonstrating the ability of the respiratory system to exert tremendous and spontaneous post-lesional neuroplasticity. One of the most studied preclinical models in respiratory insufficiency induced by cervical spinal injury is the C2 injured rat [2–8]. The initial spinal injury, performed at the cervical 2 segment induces a diaphragm paralysis due to a disruption of the main descending respiratory pathways which connect to the phrenic motoneuron pool. The contralateral side remains intact and allows animal to recover. This particular model expresses some spontaneous neuroplasticity leading to a partial reactivation of the ipsilateral phrenic and diaphragm activities. This partial recovery is sustained by activation of the silent spared contralateral bulbospinal respiratory pathways that cross the midline at the segmental level C3-C6 after a short post-lesional delay [7, 9]. These pathways are called the crossed phrenic pathways (CPP) [2, 10]. However, this reactivation is marginal and does not directly participate in the animal ventilation [11]. To date, only invasive techniques have been used to evaluate the excitability and reorganization of such pathways in vivo at various post-lesional delays. Transcranial magnetic stimulation (TMS) is a promising technique consisting of applying a brief and high intensity magnetic field at supraspinal level with the aim to depolarize the cortical and subcortical areas to elicit volleys of action potentials, which propagate to the phrenic motoneuron and diaphragm. Supraspinal connections from cortical and bulbar neurons innervating the phrenic motoneuron pool have been demonstrated in cats [12], rats [13], and humans [14, 15]. TMS in humans has been proven to be effective in the study of diaphragmatic motor evoked potential (MEP) [16–19]. Recently, specific diaphragmatic MEP (MEPdia) has been recorded following a single pulse of TMS with a large figure-of-eight coil on a respiratory insufficiency model in adult male Sprague-Dawley rats [20]. This non-invasive technique allows the assessment of the respiratory supraspinal excitability and plasticity in the rat. In the present study, using a preclinical animal model of respiratory insufficiency induced by a cervical spinal injury, we proposed to investigate 1) the early reorganization of the silent crossed phrenic pathway excitability by TMS single pulse 1 hour and 7 days post-injury, and 2) the potential correlations of the MEPdia with the remaining spared pathways and/or the functional outcome from the contralateral intact side. This novel non-invasive technique could be used as a diagnosis tool to evaluate the remaining functional spinal fibers spared by the initial injury and the putative functional prognosis of respiratory recovery.

## Materials and Methods

### Ethics statement

All experiments reported in this manuscript conformed to policies laid out by the National Institutes of Health (USA) in the Guide for the Care and Use of Laboratory Animals. These experiments were performed on 2 months-old male Sprague—Dawley rats (Janvier, France). The animals were dual-housed in individually ventilated cages in a state-of-the-art animal care facility (2CARE animal facility, accreditation A78-322-3, France), with access to food and water *ad libitum* with a 12h light/dark cycle. The Ethics committee of the RBUCE-UP Chair of Excellence (University of Paris-Sud, grant agreement No. 246556) and the University of Versailles Saint-Quentin-en-Yvelines approved these experiments.

### Chronic C2 hemisection

Tramadol (analgesic, 15 mg/kg), carpofen (anti-inflammatory, 5 mg/kg), enrofloxacin (antibiotics, 4 mg/kg), and medetomidine (100 μg/kg) were administered sub-cutaneously 10–20 mn before inducing isoflurane anesthesia in a closed chamber (in 100% O_2_). Rats were intubated and ventilated with a rodent ventilator (model 683; Harvard Apparatus, South Natick, MA, USA). Anesthesia was maintained throughout the procedure (1.5–2% Isoflurane in 100% O_2_). Skin and muscles were retracted, and a C2 laminectomy and durotomy were performed. The spinal cord was sectioned unilaterally just caudal to the C2 dorsal roots with micro-scissors followed with a micro-scalpel to ensure the section of all the remaining fibers, as described previously [5]. We deliberately chose not to do a complete hemisection, but a graded section on the injured side to leave enough ventral white matter. The wounds and skin were sutured closed. Sham rats underwent the same procedures without hemisection. Atipamezole (400 μg/kg, intra muscular) was given to reverse medetomidine. The Isoflurane vaporizer was turned off, the endotracheal tube was removed, and the rats were monitored throughout recovery. Rats received analgesic (tramadol, 15 mg/kg), antibiotics (enrofloxacin, 4 mg/kg) and anti-inflammatory (carpofen, 5 mg/kg) drugs for 2 days post-surgery.

### Transcranial magnetic stimulation (TMS)

TMS was performed using the magnetic stimulator MAGPRO X100 (Magventure, Farum, Denmark) connected to a figure-of-eight coil (CB60; dimensions: 165x85x20 mm) delivering a unique biphasic pulse (380 μs in duration) with an intensity of the stimulus expressed as a percentage of maximum output of the stimulator (% MO). The MO was set-up at 90%, and the center of the coil placed at -6 mm from Bregma. This position is known to elicit a specific diaphragmatic Motor Evoked Potential (MEPdia) from supraspinal stimulation transiting through the spinal cord in rats [20]. Each animal received about 100 TMS single pulses across the different experimental conditions. As described previously [20], interpulse duration was always above 10s to avoid low frequency repetitive TMS like effects.

### Electrophysiological recordings

A total of 39 animals were used in this study for terminal electrophysiological recordings. They were randomly divided into 3 different groups: Sham (n = 21), 1 hour post-injury (1h P.I. n = 10) and 7 days post-injury (7d P.I. n = 8). As described previously [20, 21], anesthesia was induced using isoflurane (3.5% in 21% O_2_ balanced) through a nose cone. A 25G catheter was placed in the tail vein. The rats were tracheotomized and pump ventilated (Rodent Ventilator, model 683; Harvard Apparatus, South Natick, MA, USA). The ventilation rate was adjusted below the animal central apneic threshold throughout the experiment. EtCO_2_ was monitored using an infrared capnograph (Viamed, VM-2500-M). Animals were placed on a heating pad to maintain a constant body temperature and their rectal temperature was monitored throughout the experiment. Arterial pressure was measured through a catheter inserted in the right femoral artery. Arterial and tracheal pressures were monitored continuously with transducers connected to a bridge amplifier (AD Instruments, Dunedin, New Zealand). Isoflurane anesthesia was then slowly converted to urethane anesthesia (1.8 g/kg, i.v.; Sigma-Aldrich). The depth of anesthesia was confirmed by the absence of any response to toe pinch. A laparotomy was performed, and the liver was gently moved caudally to access the diaphragm. Gauze soaked with warm phosphate-buffered saline was placed on the liver to prevent dehydration. The head of the animal was placed on a non-magnetic custom-made stereotaxic apparatus which allowed its positioning from the center of the figure-of-eight coil to -6 mm from Bregma, at an angle of 0°, as shown previously [20]. Two custom-made hooked bipolar electrodes were placed on each mid-costal part of the diaphragm and left there for the entire duration of the experiment. Diaphragm spontaneous poïkilocapnic normoxic or transient mild asphyxia (by pinching the animal nose for 15 s) EMGs and MEPdia induced by a single pulse of TMS were amplified (gain, 1k; A-M Systems, Everett, WA, USA) and band pass-filtered (100 Hz to 10 kHz). The signals were digitized with an 8-channel Powerlab data acquisition device (Acquisition rate: 100 k/s; AD Instruments, Dunedin, New Zealand) connected to a computer and analyzed using LabChart 7 Pro software (AD Instruments, Dunedin, New Zealand). The bilateral diaphragmatic EMGs were integrated (50 ms decay).

At the end of the experiment (around 3 hours under urethane anesthesia), animals were euthanized with an i.v. overdose of urethane.

### Histological reconstruction of the extent of C2 injury

Immediately after euthanasia, the cervical spinal cord was dissected (C1 to C4) and placed overnight in cold 4% paraformaldehyde (in 0.1 M Phosphate Buffered Saline). The segment was then cryoprotected in ascending concentration of sucrose solution and frozen. Transverse sections (30 μm thickness) were collected and the injury was assessed with cresyl violet staining. A light microscope was used to draw the extent of the injury and the latter was reported on a stereotaxic transverse plane as described previously [5, 6]. Each injury was digitized and analyzed with Image J software (NIH). The percentage of spared ventral white matter on the injured side was calculated by reference to a complete hemisection (which is 0% of spared white matter, as previously described [22]).

### Data processing

The amplitude and frequency of 10 integrated diaphragm EMG inspiratory bursts during normoxia and mild asphyxia was calculated for each animal from the injured and intact sides with LabChart 7 Pro software (AD Instruments).

The average of at least 5 MEPdia was calculated with LabChart Pro software (AD Instruments). The baseline-to-peak amplitude of the first negative wave (N1) of each averaged MEPdia was measured. MEPdia latency was defined as the first electrical (positive or negative) deviation following the magnetic pulse artefact (as previously described in rodent TMS studies [20, 23]).

Normality of the data distribution was assessed using a Kolmogorov-Smirnov test and log transformation was performed when required. Two way ANOVA with Bonferoni correction for multiple comparisons was performed between animal groups followed by post-hoc Student t-test. A Student paired t-test was performed to demonstrate the reorganization of the MEPdia between both sides (injured vs. intact) in the same animal at various post-injury delays (1 hour and 7 days). All the data are presented as mean ± SEM. Individual values of the MEPdia amplitude are represented as well as the mean value to highlight the difference between the intact and injured side in the same animal. A test was considered significant if p<0.05. A linear regression has been performed to compare the MEPdia, diaphragm contralateral activity and remaining spared white matter. Regression coefficient R^2^ and p values are indicated in the figure legend.

## Results

### Physiological effects of a C2 injury

The body weight of the animal groups was similar for Sham animals and 1h post-injury (sham: 380±51g, 1h P.I.: 398±13g). However, at 7d P.I., the body weight declined significantly (342±8g) compared to Sham and 1h P.I. groups. The body temperature increased significantly in 1h P.I. animals compared to sham and 7d P.I. animals (37.8±0.2°C compared to 36.7±0.1°C and 36.8±0.2°C respectively). End-tidal CO_2_ was similar in all groups (30±0.6 mmHg for sham, 29.6±3 mmHg for 1h P.I. and 30±1 mmHg for 7 d P.I.). Mean arterial blood pressure (MAP) was maintained throughout the experiment for all groups (MAP start: 92±2 mmHg, MAP End: 99±3 mmHg for sham; MAP start: 70±7 mmHg, MAP End: 68±7 mmHg for 1h P.I.; MAP start: 97±5 mmHg, MAP End: 96±4 mmHg for 7d P.I.). However, the corresponding MAP start and MAP end decreased significantly in 1h P.I. animal group compared to sham and 7d P.I. (Table 1).

**Table 1:** Physiological parameters.

	Body weight (g)		Temperature (°C)		EtCO2 (mmHg)		MAP Start (mmHg)		MAP End (mmHg)	
Treatment Group	Mean	SEM	Mean	SEM	Mean	SEM	Mean	SEM	Mean	SEM
Sham	380	51	36.7	0.1	30	0.6	92	2	99	3
1h C2HS	398	13	37.8^b^	0.2	29.6	3	70^b^	7	68^b^	7
7d C2HS	342^a^	8	36.8	0.2	30	1	97	5	96	4

MAP: Mean arterial pressure, EtCO2: End-tidal CO2,

^a^ p<0.05 compared to Sham and 1h,

^b^ p<0.05 compared to Sham and 7d.

### Effect of a C2 injury on diaphragm activity

**Fig 1:**
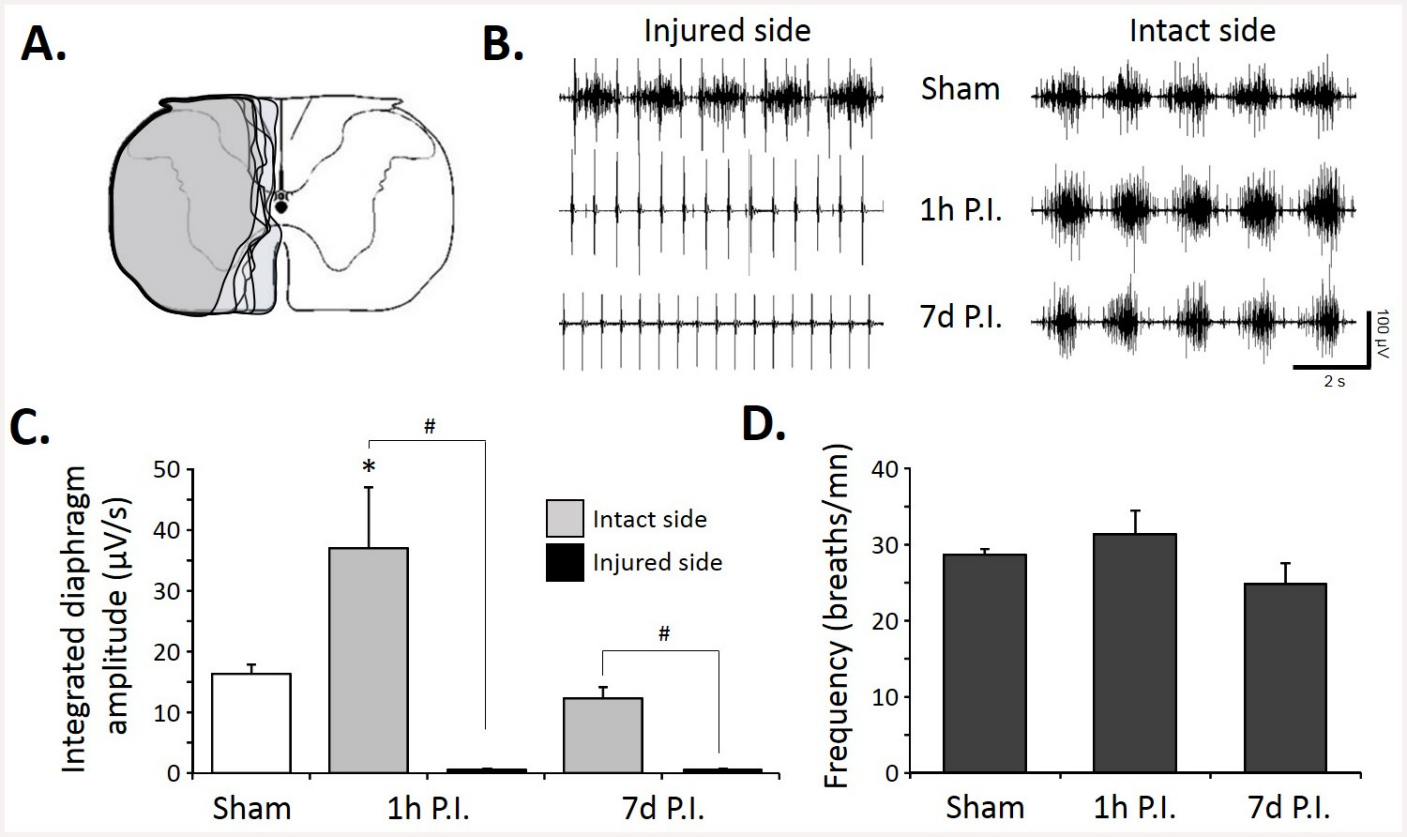
Diaphragm activity following C2 spinal cord injury. A. Representative extent of injury in 8 animals at 7 days post-injury. B. Representative traces of raw diaphragm EMG for Sham, 1 hour post-injury (1 h P.I.) and 7 days post-injury (7 d P.I.). Note the absence of rhythmic activity on the injured side at 1h and 7d P.I. The deflexion of the signal recorded on the injured side is due to the parasitic recording of the electrocardiogram. C. Integrated diaphragm amplitude for Sham, 1h P.I. and 7d P.I. animals. Note the absence of diaphragm activity on the injured side for 1h and 7d P.I. groups. D. Diaphragm frequency in poïkilocapnic breathing Sham, 1h and 7d P.I. animals. * p<0.05 compared to Sham and 7d P.I. groups; # p<0.05 between sides (injured vs. spared side), two-way ANOVA with Bonferonni correction tested.

**Fig 2:**
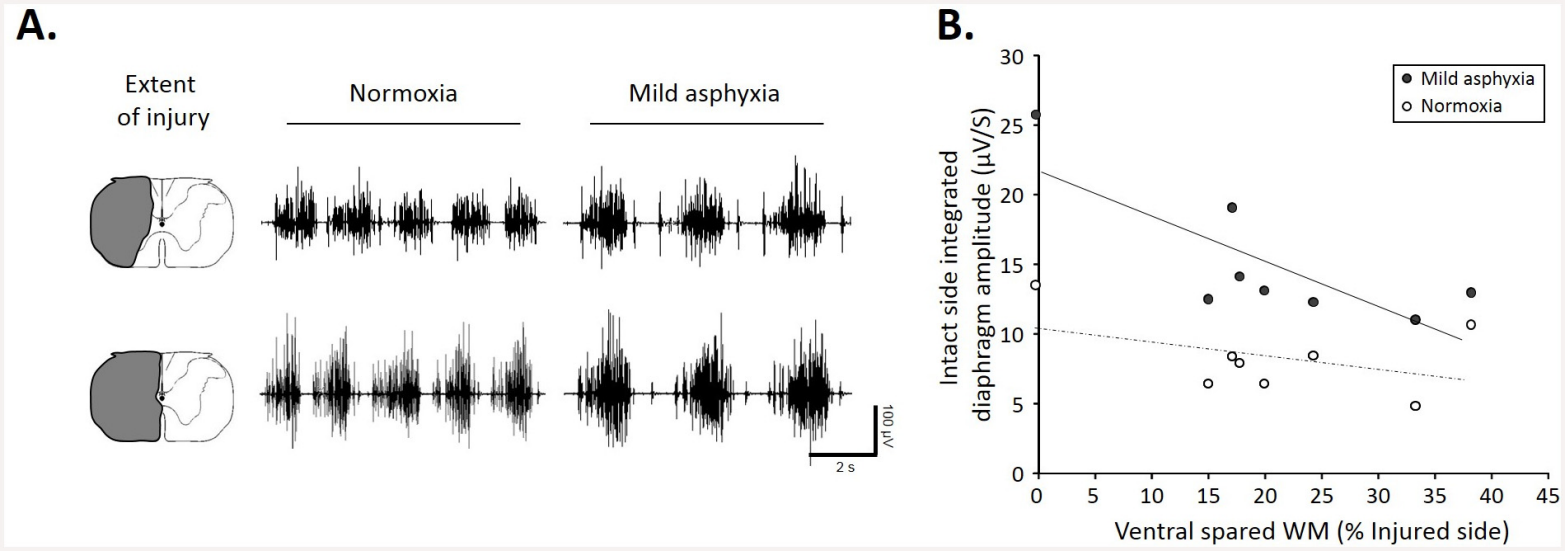
Correlation between diaphragm activity and extent of C2 injury in normoxic and mild asphyxic rats. A. Representative EMG recording during normoxia and mild asphyxia with corresponding extent of injury. Note a greater EMG amplitude with less spared white matter. B. Correlation between integrated contralateral diaphragm amplitude and ventral spared white matter (during normoxia (white dots, R^2^:0.138, p = 0.011) and mild asphyxia (black dots, R^2^:0.609, p<0.001)). Note higher contralateral diaphragm amplitude with smaller spared white matter during mild asphyxia.

The size of the C2 injury was similar in the lateral part in all injured animals (7d P.I.). We deliberately chose not to perform a complete hemisection and left some ventro-medial part of the spinal cord, allowing some spared ventral white matter (Fig 1A). A lateral C2 injury was sufficient to abolish the diaphragmatic activity on the injured side at 1h P.I., and it persisted at 7d P.I. (Fig 1B). The diaphragmatic electromyogram on the intact side did not show any difference in the frequency and amplitude in all groups (Fig 1B). Quantitatively, sham group showed an integrated diaphragm average amplitude of 16.3±1.5 μV/s, similar to the intact side of the 7d P.I. group (12.3±1.8 μV/s) whereas at 1h P.I., a statistically significant increase in diaphragm integrated amplitude emerged compared to the other groups in the intact side (36.9±10 μV/s, Fig 1C). No diaphragm activity was observed at 1h and 7d P.I. on the injured side in normoxic poïkilocapnic breathing and during mild asphyxia (Fig 1C, data not shown for the mild asphyxia). Similar respiratory frequencies were recorded among these groups during normoxic poïkilocapnic breathing (sham: 28.1±0.8 breaths/mn; 1h P.I.: 31.4±3 breaths/mn; 7d P.I.: 24.8±2.7 breaths/mn). The diaphragm EMG amplitude during normoxia and mild asphyxia correlated strongly with the ventral spared white matter (Fig 2A). Interestingly, less ventral spared white matter led to a larger contralateral diaphragm activity during normoxia and mild asphyxia (Fig 2B), whereas no ipsilateral diaphragm activity was recorded in our animals in both conditions (not shown).

### Effect of a TMS pulse on the MEPdia

**Fig 3:**
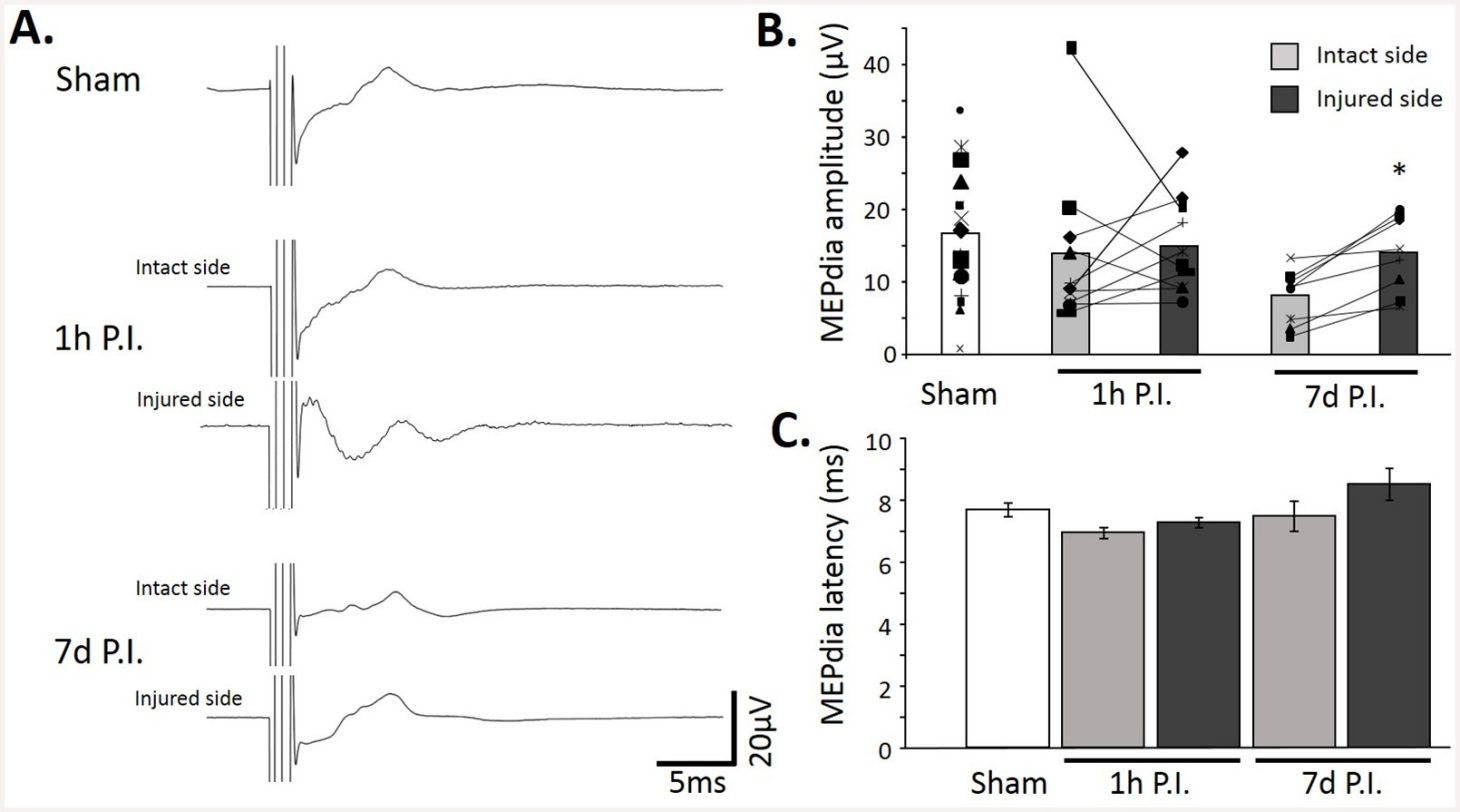
Diaphragm motor evoked potentials in sham, 1h P.I. and 7d P.I. animals. A. Representative MEPdia recordings for sham, 1h P.I. spared and injured sides and 7d P.I. spared and injured sides. Note the diminution of the MEPdia amplitude in the spared side compared to the injured side at 7d P.I. B. MEPdia amplitude in Sham, 1h P.I. and 7d P.I. animals. Note the reorganization of the MEPdia at 7d P.I. (increased in the injured side compared to the spared side) when compared to 1h P.I. group. C. MEPdia latency in sham, 1h P.I and 7d P.I. group. Note the trend of higher latency in the 7d P.I. injured side group. * p<0.05 compared to spared side.

A TMS single pulse (90% MO) at -6 mm over the Bregma in a urethane anesthetized sham animal induced an ample MEPdia on both sides of the diaphragm (Fig 3A). Surprisingly, at 1h and 7d P.I., a MEPdia could be recorded on both the injured and the intact side of the diaphragm in each animal (Fig 3A, S1 Supporting Information). Comparable average MEPdia amplitude was recorded in all groups (Sham: 16.4±2 μV; 1h P.I.: Intact side = 13.9±3.6 μV; Injured side = 14.8±2.2 μV; 7d P.I.: Intact side = 8.1±1.4 μV; Injured side = 13.8±1.9 μV) with a non-significant trend indicating amplitude reduction on the intact side in 7d P.I. group. No difference side by side in the amplitude of MEPdia was observed in sham animals (paired t-test, two-tailed p value = 0.191, data not shown), and we decided to average the amplitude of each side and present the data per animal. However, when 1h P.I. and 7d P.I. groups were compared individually side by side (intact side vs. injured side), a significant increase in MEPdia amplitude was observed at 7d P.I. for each animal at the injured side compared to the intact side (paired t-test, two-tailed p value = 0.0022) whereas no significant difference was observed at 1h P.I. (paired t-test, two-tailed p value = 0.803) (Fig 3B). MEPdia latencies were similar in all groups (Sham: 7.7±0.2 ms; 1h P.I.: Intact side = 6.9±0.2 ms; Injured side = 7.2±0.2 ms; 7d P.I.: Intact side = 7.5±0.5 ms; Injured side = 8.5±0.5 ms) (Fig 3C).

### Correlation among the injury size, MEPdia, and contralateral diaphragm activity

**Fig 4:**
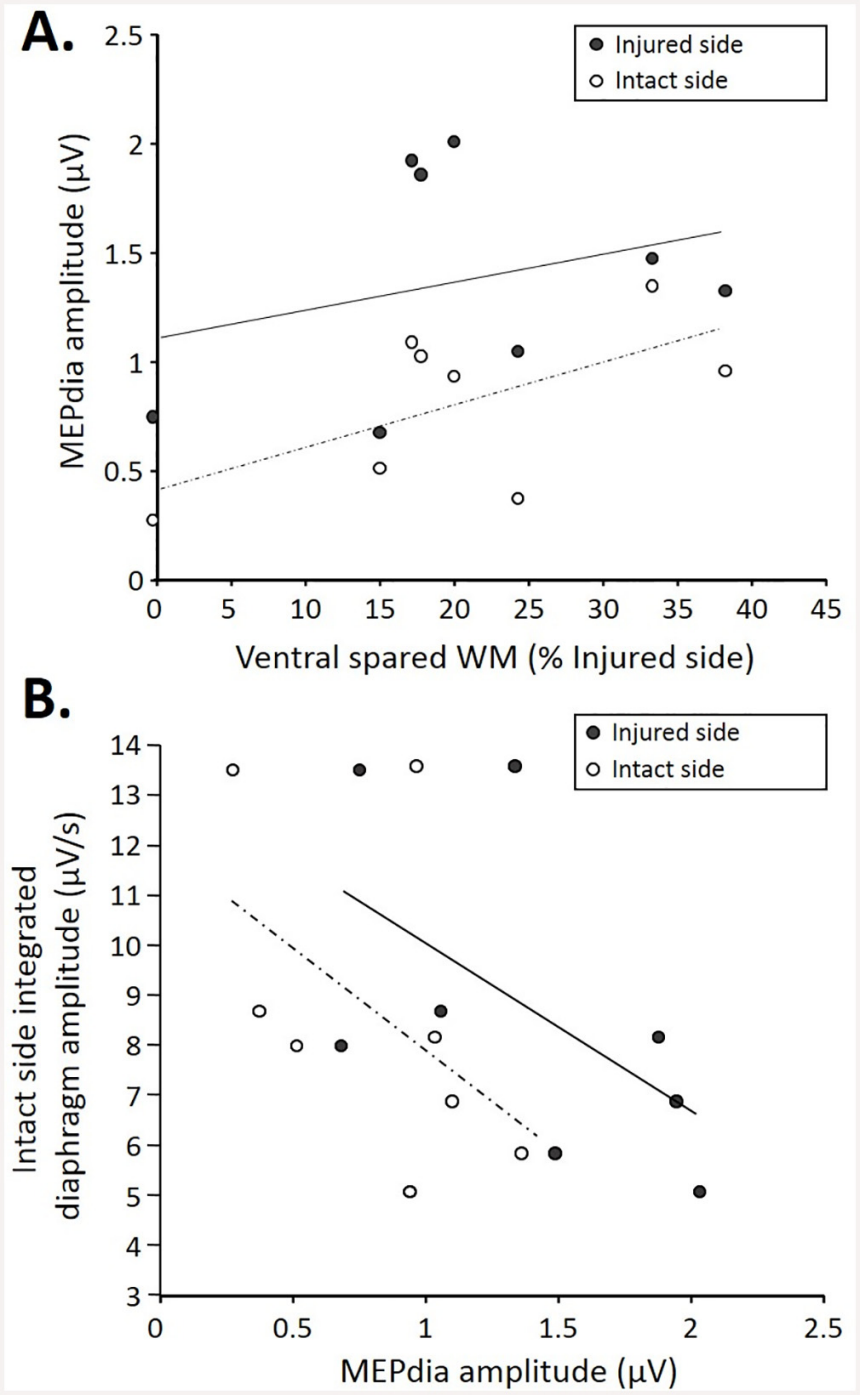
Correlations among MEPdia amplitude, extent of injury, and contralateral diaphragm activity at 7d P.I. A. Correlation of the extent of injury (represented as the ventral spared white matter) with MEPdia amplitude on the injured (black dots, R2:0.16, p = 0.034) and spared (white dots, R2:0.364, p = 0.149) side. Note the higher MEPdia recording with bigger spared white matter. B. Correlation between contralateral diaphragm amplitude and MEPdia amplitude on injured (black dots, R2:0.559, p = 0.005)/spared (white dots, R2:0.495, p = 0.014)sides. Note higher diaphragm EMG with lower MEPdia amplitude.

As expected, the injuries extents in 7d P.I. animal group varied from 0% to 38% of ventral spared white matter (Fig 4A, x axis). A correlation was observed between both intact and injured MEPdia amplitudes and the ventral spared white matter (express as a percentage of the injured side) at 7d P.I. More ventral spared white matter led to a higher recorded MEPdia induced by TMS in both injured and intact sides. For a given ventral spared white matter value, the MEPdia from the injured side was systematically higher compared to that of the intact side (Fig 4A). The intact side integrated diaphragm amplitude during poïkilocapnic normoxic breathing in 7d P.I. group correlated with the recorded MEPdia amplitude in the injured and intact sides. Higher intact side diaphragmatic integrated amplitude corresponds to a lower MEPdia whereas a lower amplitude in integrated diaphragm recording corresponded to a higher MEPdia recorded in the intact side. Similar trends were observed when comparing the MEPdia obtained from the injured side with the intact side integrated diaphragm amplitude (Fig 4B). The MEPdia amplitude induced by a TMS pulse could predict the amplitude of diaphragmatic activity on the intact side 7d P.I.

## Discussion

This study demonstrated for the first time the use of a non-invasive transcranial magnetic stimulation technique, in the assessment of the phrenic motoneuron excitability in a preclinical model of respiratory insufficiency. These results support the feasibility of using TMS to study respiratory dysfunction associated with neurological injury and disease.

**Fig 5:**
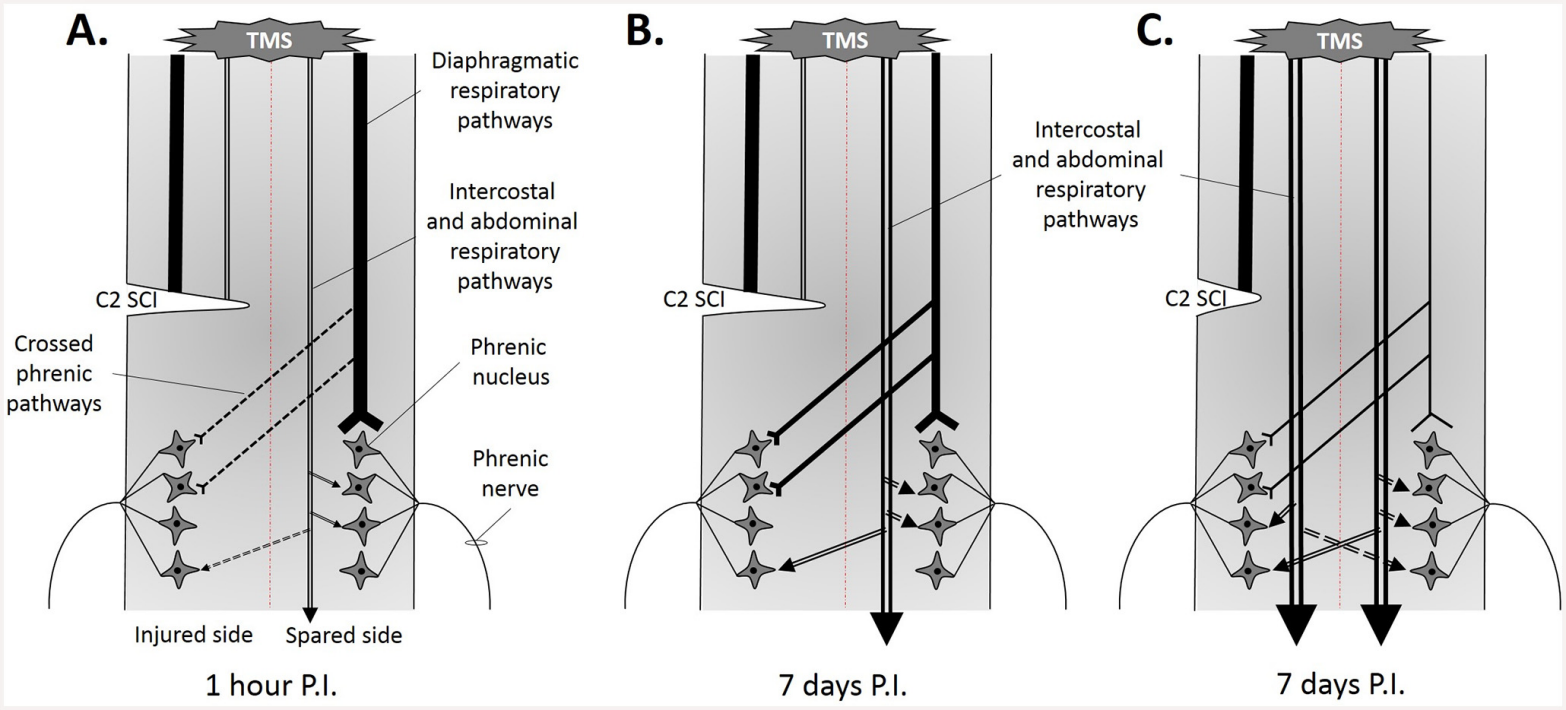
Proposed model of the correlation of early respiratory bulbospinal descending pathways rewiring with extent of injury. A. Representation of the bulbospinal descending tract 1h P.I. The contralateral pathways remain intact and allow the transit of the MEPdia induced by a single TMS pulse. Crossed phrenic pathways can also be activated with TMS. B. Early reorganization of bulbospinal respiratory pathway following a large C2 injury. The evoked potential by TMS transit through less spared fibers, allowing a smaller recorded MEPdia on the ipsilateral and contralateral sides (less connections from respiratory related fibers to the contralateral side). C. Reorganization of respiratory bulbospinal descending tracts with a partial lateral C2 section. More respiratory related tracts are spared and activated with TMS, inducing a bigger MEPdia. However, these pathways remain silent when the animal breathes by itself, suggesting a potential neuroanatomical substrate for inducing functional respiratory recovery.

Interestingly, following a cervical spinal partial cord injury at C2, a MEPdia can be recorded after a TMS single pulse stimulation on both sides at 1h and 7d P.I. despite the absence of diaphragmatic spontaneous activity during poïkilocapnic and transient asphyxia breathing. TMS transits induced the volleys of descending action potentials supraspinally through the contralateral bulbospinal main respiratory pathways, and these volleys crossed the midline at the segmental level C3-C6 (Fig 5A). Despite the fact that these crossed phrenic synapses were not spontaneously active immediately after the acute C2 injury, a MEPdia could be recorded on the side of injury, suggesting the presence of pre-existing silent synaptic crossed connections, which could be revealed by TMS. An evoked response induced by a contralateral C1 intraspinal stimulation could also be recorded on the ipsilateral side following a chronic C2 injury at a longer time post-injury [24, 25]. However, no differences in the evoked crossed phrenic pathways between 2 weeks and 4 weeks post-injury have been demonstrated [25], suggesting that spinal plasticity occurs earlier. Indeed, a profound morphological reorganization of these crossed phrenic pathways, especially an increase in dendrodendritic appositions and synaptically active zones in the ipsilateral phrenic motor nucleus could be observed as early as 2h post-C2 injury [26] (see Fig 5B and 5C). These anatomical events could be a part of the neuronal substrate to sustain the MEPdia evoked by TMS at 1h post-injury on the injured side. Moreover, interconnections between the ipsilateral and contralateral phrenic motoneurons as well as projections from the rostral ventral respiratory group with contralateral crossed connections to the injured part at the segmental level could also be involved in the activation of the crossed phrenic pathways and potentially activated after acute and chronic injured animals [27]. A profound and spontaneous reorganization of descending respiratory bulbospinal pathways was observed following C2 injury, contributing to the functional spontaneous restoration of the diaphragmatic activity at longer post-lesional delays [28]. In this present study, we chose to study the early reorganization and changes in spinal excitability following C2 injury. The lack of difference in MEPdia amplitude in Sham and 1h P.I. group observed in our study could be explained by the fact that the C2 initial injury induces a spinal shock at the injured side and an immediate massive release of glutamate from the axonal stubs projecting to the deafferented phrenic motoneurons. The massive amount of glutamate is excitotoxic and modifies the intraspinal excitability on and around the phrenic motoneurons, probably due to the removal of inhibitory connections and/or hyperexcitability of the spinal stub [20]. This could contribute to the hyperexcitability of the crossed phrenic pathways observed 1h after the injury. An interesting observation is that the latency of MEPdia evoked by TMS single pulse is slightly diminished 1h P.I. compared to Sham and 7d P.I., suggesting a change in spinal excitability in favor of the signal transit. Similar MEPdia diminished latencies have been recorded in response to a TMS single pulse applied at the spinal stub following a complete C2 transection [20]. The absence of significant differences in MEPdia latencies across groups and sides suggests that the number of synapses, which the signal transits through from stimulus location (-6 mm from Bregma), and the response recording in the diaphragm are similar. However, following a C2 hemisection, spinal interneurons participate in spinal plasticity following the lesion. They could modulate the crossed phrenic pathways and the respiratory descending activities [29], which may explain longer latency observed at 7d P.I. in our study. At longer P.I. time (weeks to months), several events take place in the injured spinal cord. Increased occupational area in activated microglia around the phrenic motoneurons at 7d P.I. and an increase in respiratory plasticity associated molecules, BDNF and its high-affinity receptor TrkB (along with its phosphorylated form pTrkb), in and around the phrenic motoneurons were observed following C2 lateral injury [30, 31]. The BDNF/TrkB cascade allows microglia to control the excitability of neuronal network and it can unmask hidden neuronal circuitry that is normally silent [32]. This can contribute to the changes in phrenic motoneuron excitability at 7d P.I. in response to TMS. Nevertheless, no difference in breathing frequency on the intact side has been observed between 1h and 7d P.I., suggesting that the plasticity occurs preferentially at the spinal segmental level instead of the brainstem respiratory structures above the injury side. We cannot exclude the fact that following a chronic C2 hemisection, profound morphological changes and reorganization occur at the diaphragm neuromuscular junction overtime [33], and they may play a role in the excitability reorganization of MEPdia following TMS single pulse at 7d P.I.

Following a cervical hemisection, the blood respiratory gazes and pH oxygenation were not affected despite the fact that one hemidiaphragm was paralyzed [34, 35]. The implications of other respiratory related muscles (i.e. intercostals and abdominals, Fig 5B and 5C) for maintaining a certain respiratory gazes exchange homeostasis and for compensating for the maintenance of respiratory function following a chronic C2 hemisection have already been demonstrated [36, 37]. Interestingly, the injury size contributes to the spontaneous functional recovery as well as to the respiratory deficit. In fact, a larger injury induces more respiratory deficits measured by plethysmograph, suggesting an effect on other respiratory related pathways in rats [22] and mice [38]. In our study, we observed a perfect inverse correlation between the extent of injury and contralateral diaphragm activity during poïkilocapnic and mild asphyxia breathing at 7d P.I. A small injury will lead to a smaller contralateral diaphragm activity, suggesting that the respiratory descending pathways to the abdominal and intercostal muscles are less affected by the initial injury and that they further contribute to the whole respiration (Fig 5C). By contrast, if the injury affects more respiratory related pathways (less spared white matter), the contralateral diaphragm will adapt its activity (bigger amplitude) to maintain the respiratory homeostasis. Another interesting point is that the CPP plays a marginal role in the tidal volume following a chronic C2 injury, suggesting that others respiratory related pathways are implicated in the generation of inspiration [11]. A lateral C2 injury is sufficient to permanently abolish the ipsilateral diaphragm activity, allowing more bulbospinal descending respiratory related spared pathways located medially in the spinal cord to re-connect the deafferented phrenic motoneurons [9]. In addition, a hyperactivity of phrenic motoneuron is observed also in supraspinal areas following a chronic C2 hemisection, suggesting the establishment of supraspinal compensatory routes which were initially inactive [39]. These increases in synaptic connectivity could contribute to the early changes in phrenic motoneuron excitability and reorganization demonstrated at 7d P.I. by TMS.

Another interesting point to discuss is the discrepancy observed between larger contralateral amplitude of MEPdia and smaller contralateral spontaneous diaphragm activity at 7d P.I. The excitability induced by TMS at the spinal level does not reflect the physiological supraspinal drive (i.e., spontaneous descending respiratory drive from the brainstem areas). This suggests that TMS recruits more supraspinal existing silent pathways connected to the contralateral phrenic motoneurons with inhibiting projections to the brainstem. These inhibiting projections from cortical areas have been observed in humans and rats [40, 41]. These pre-existing anatomical pathways could be the targets for innovative therapeutics to enhance the respiratory function following a C2 injury.

To conclude, TMS could be used as a functional diagnostic tool to investigate the remaining silent and functional spared pathways and it could be used to assess the reorganization of synaptic excitability following a cervical spinal cord injury as well as new therapeutics.

## Supporting Information

S1 Supporting Information Individual traces (diaphragm activities, MEPdia) for each animal included in this study. (PDF)
