# Supplementary material for: Reorganization of Respiratory Descending Pathways following Cervical Spinal Partial Section Investigated by Transcranial Magnetic Stimulation in the Rat
Source: PLoS One. 2016 Feb 1;11(2):e0148180. doi: 10.1371/journal.pone.0148180 (PMC4734706; doi:10.1371/journal.pone.0148180)

Individual raw values of diaphragm activity and MEP dia (average of 5 to 10 single MEP)

Sham animals

Rat #52

Ipsi | Contra

Diaphragm activity

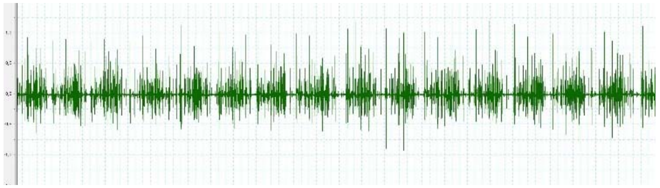

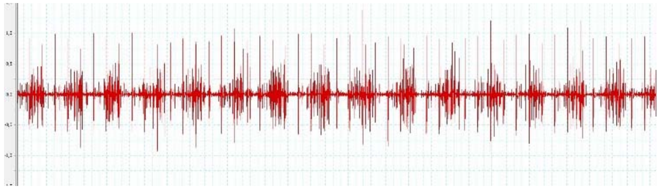

MEP dia

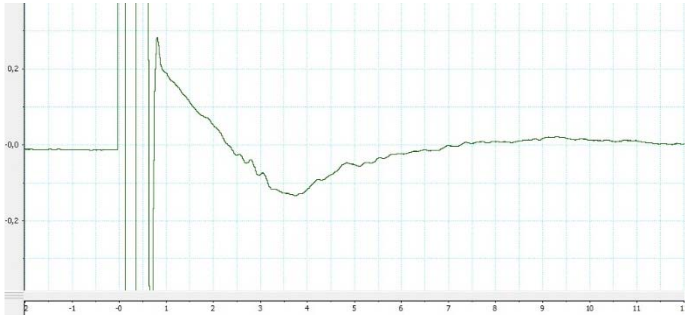

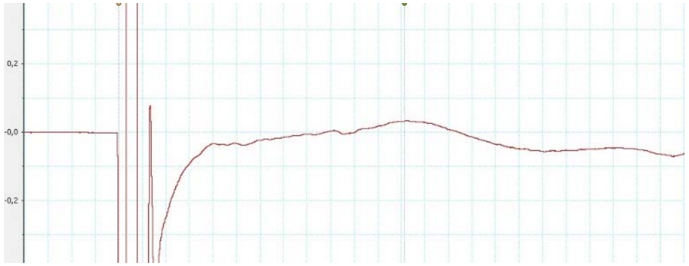

Sham animals

Rat #53

Ipsi | Contra

Diaphragm activity

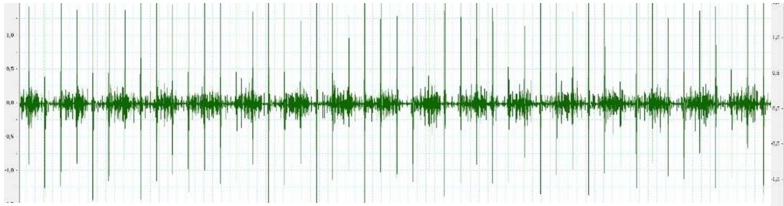

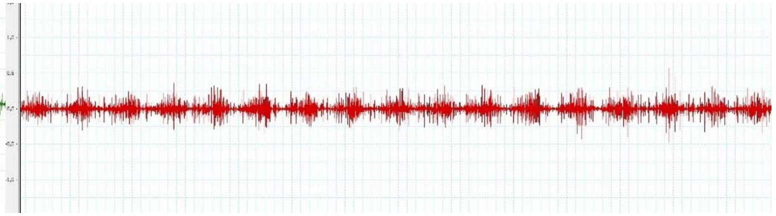

MEP dia

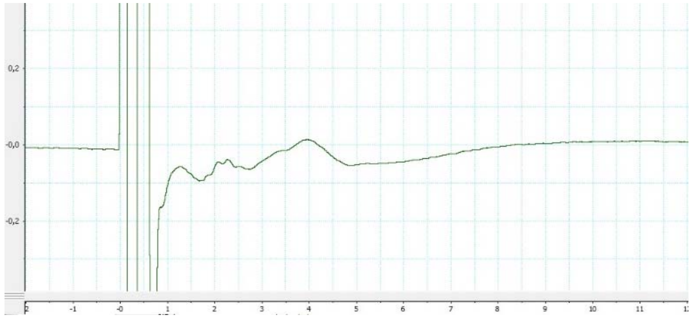

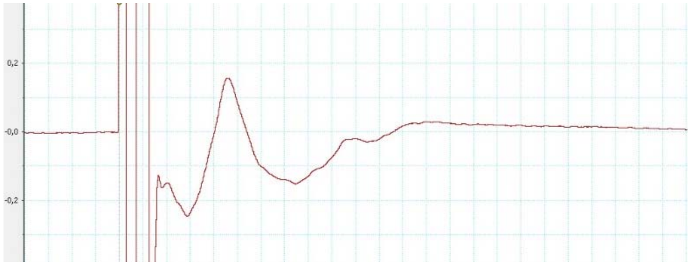

Sham animals

Rat #54

	Ipsi	Contra
Diaphragm activity	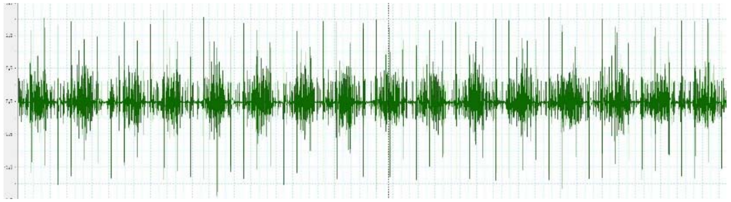	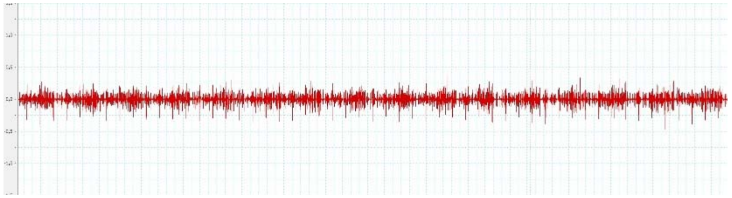
MEP dia	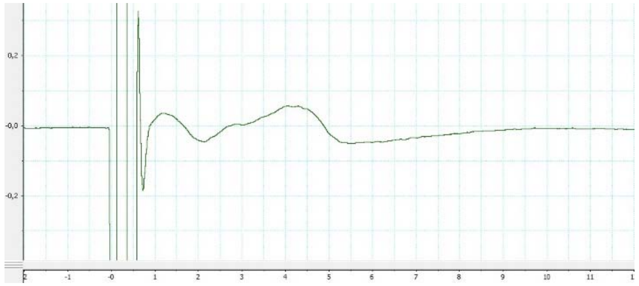	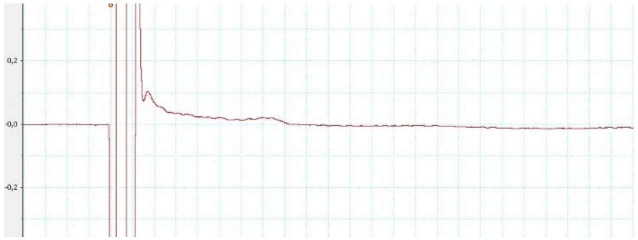

Sham animals

Rat #56

Ipsi | Contra

Diaphragm activity

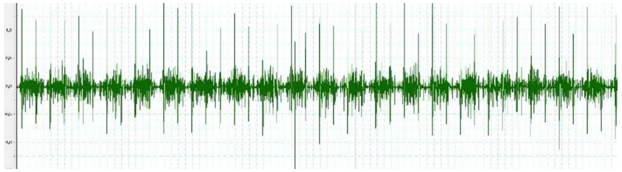

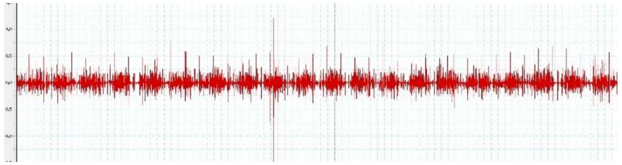

MEP dia

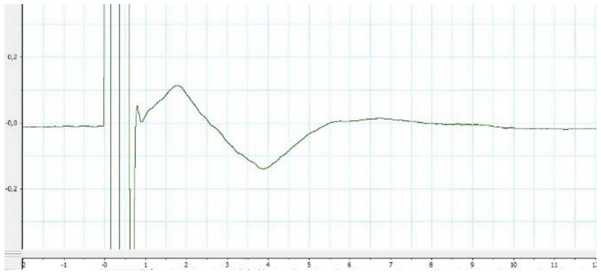

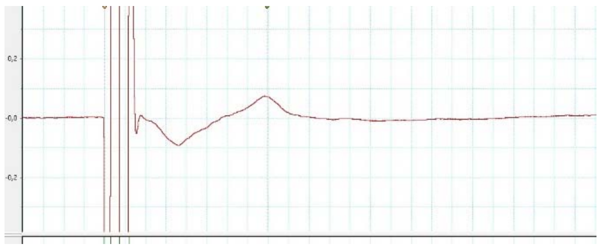

Sham animals

Rat #57

Ipsi

Contra

Diaphragm activity

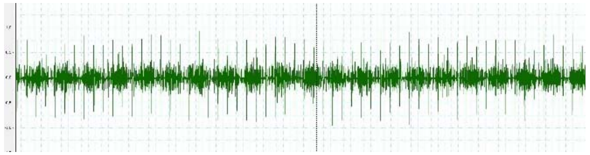

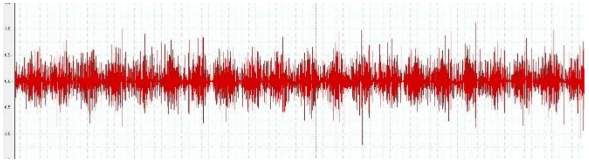

MEP dia

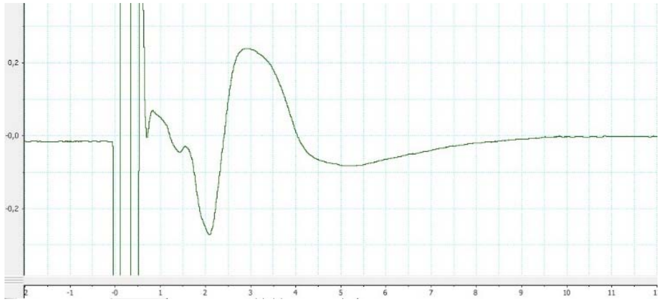

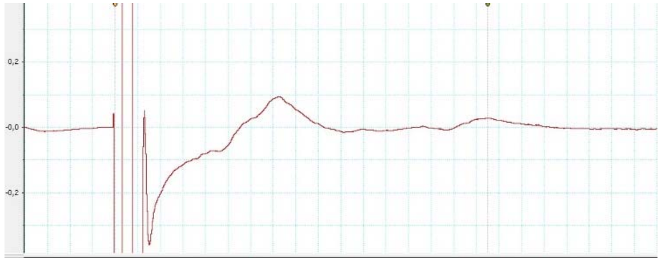

Sham animals

Rat #58

Ipsi

Contra

Diaphragm activity

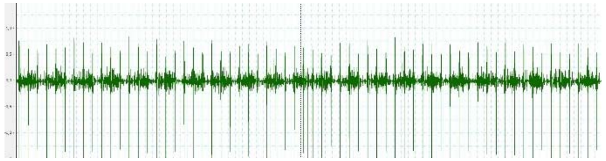

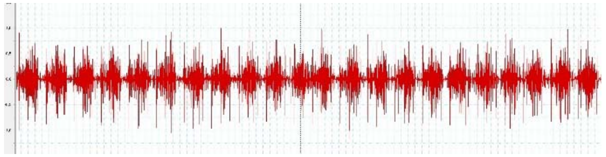

MEP dia

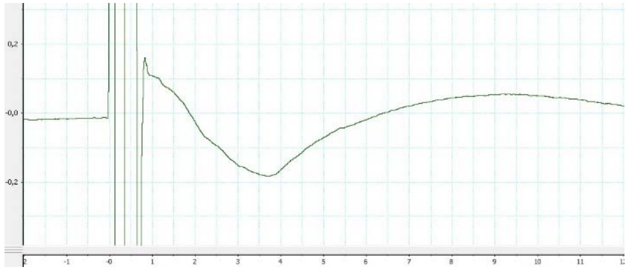

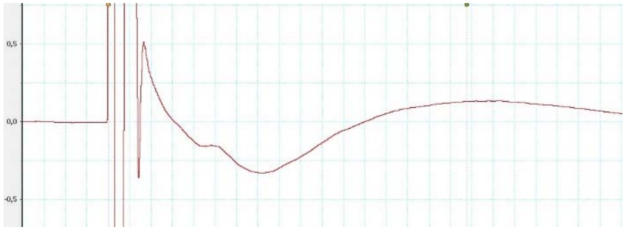

Sham animals

Rat #59

	Ipsi	Contra
Diaphragm activity	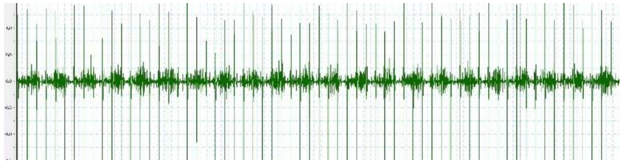	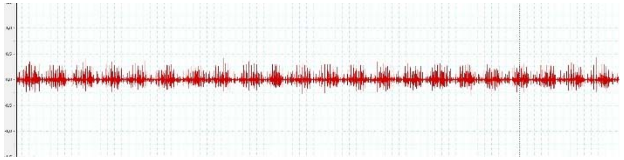
MEP dia	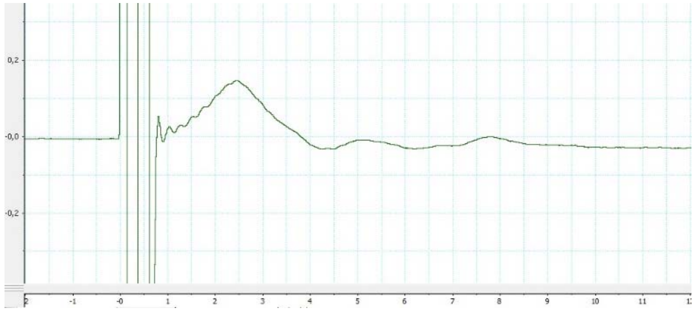	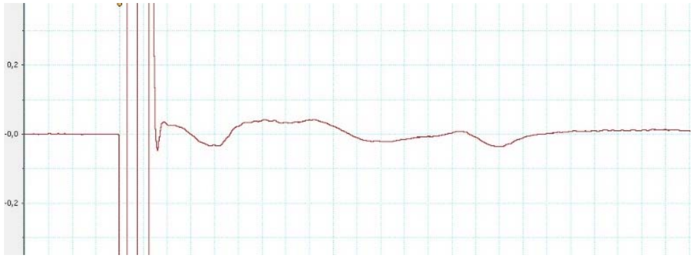

Sham animals

Rat #60

	Ipsi	Contra
Diaphragm activity	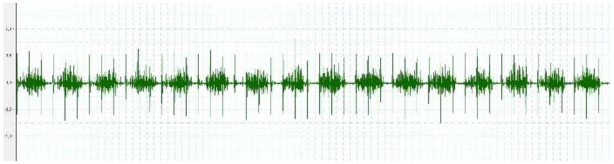	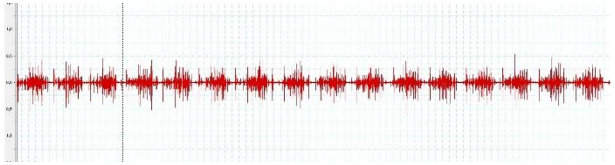
MEP dia	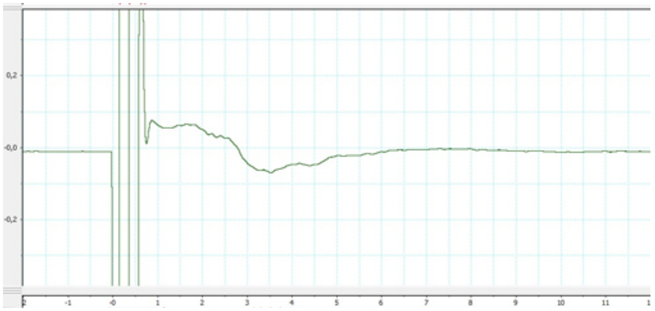	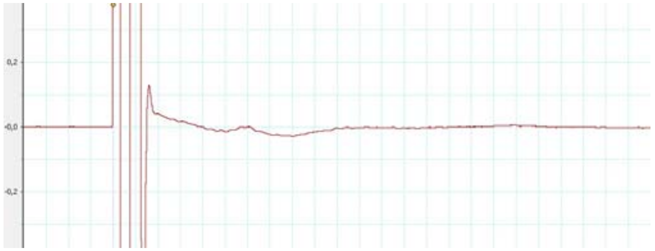

Sham animals

Rat #61

Ipsi

Contra

Diaphragm activity

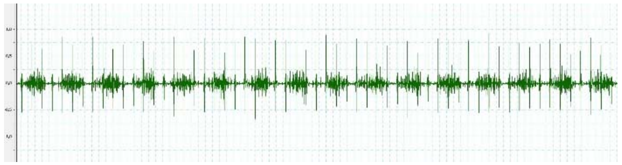

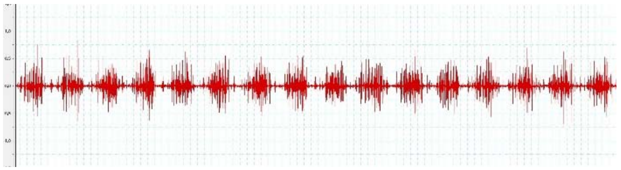

MEP dia

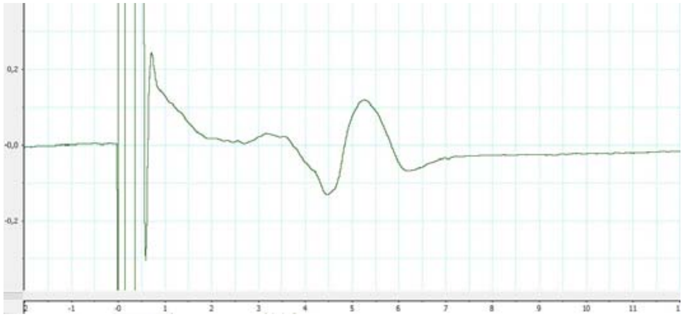

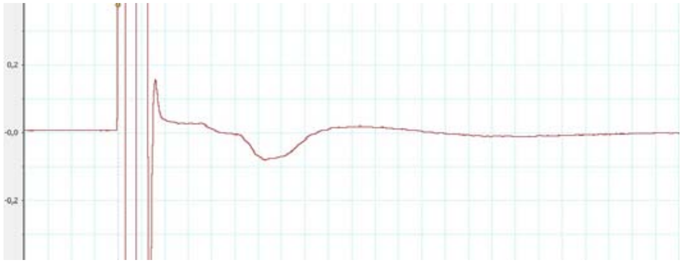

Sham animals

Rat #62

Ipsi | Contra

Diaphragm activity

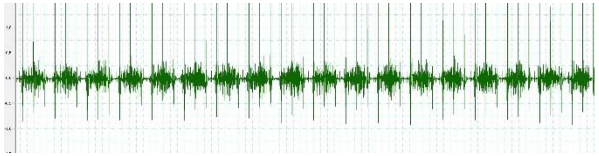

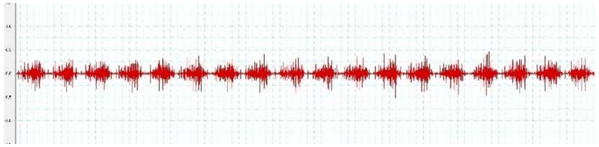

MEP dia

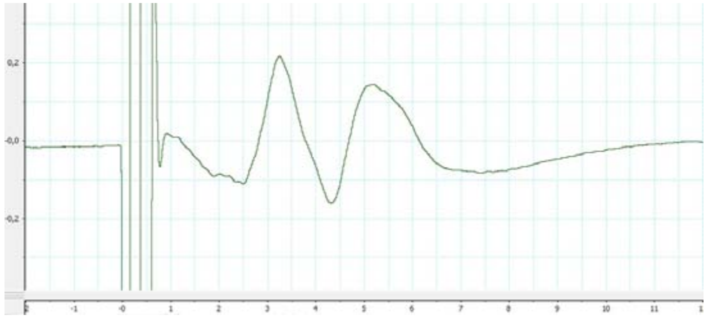

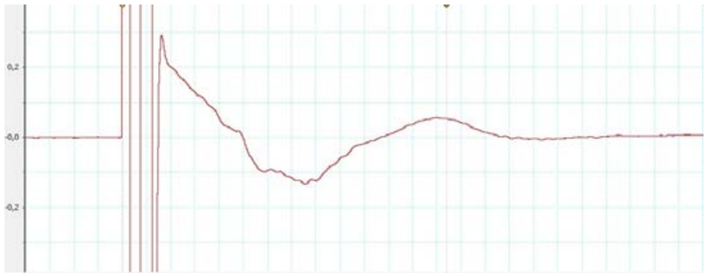

Sham animals

Rat #63

Ipsi

Contra

Diaphragm activity

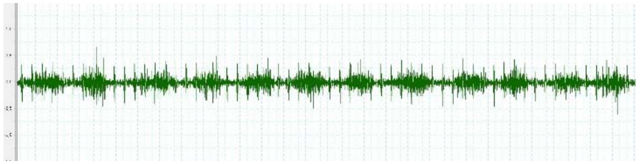

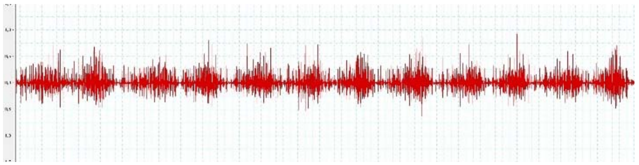

MEP dia

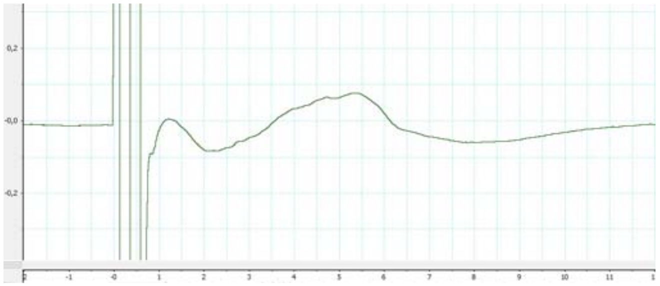

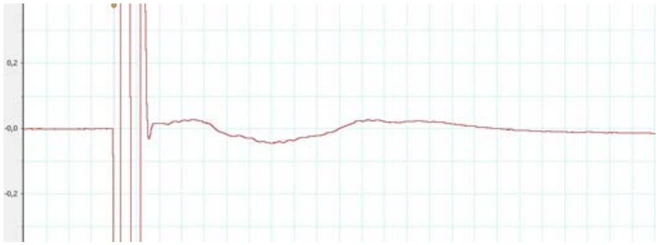

Sham animals

Rat #64

Ipsi

Contra

Diaphragm activity

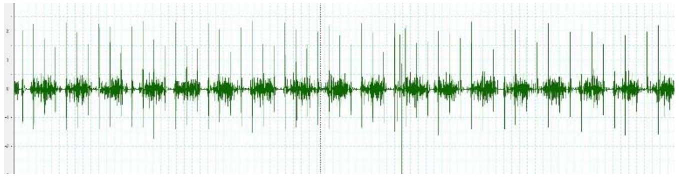

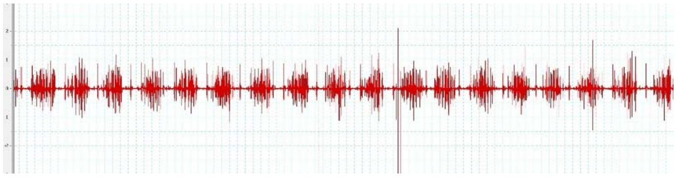

MEP dia

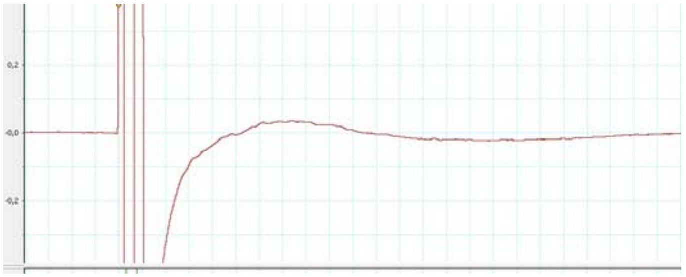

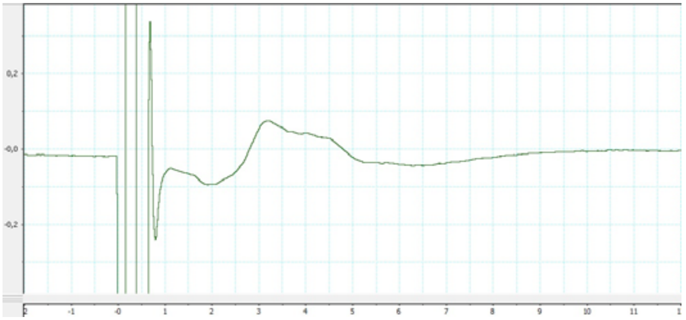

Sham animals

Rat #65

Ipsi

Contra

Diaphragm activity

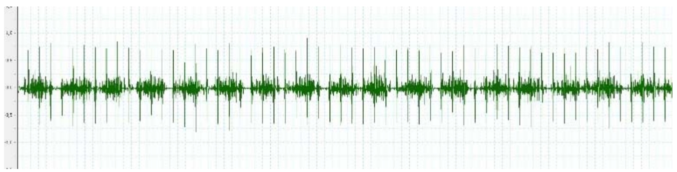

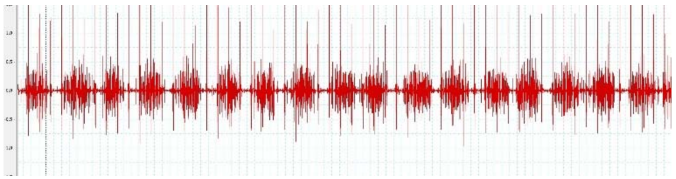

MEP dia

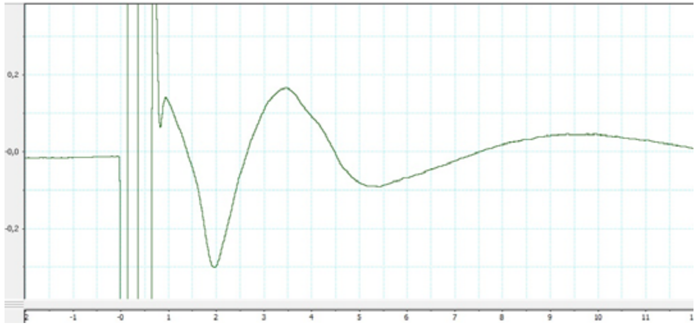

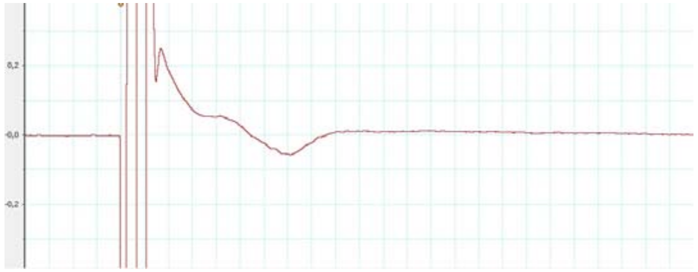

Sham animals

Rat #42

Ipsi

Contra

Diaphragm activity

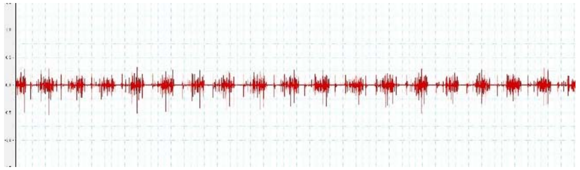

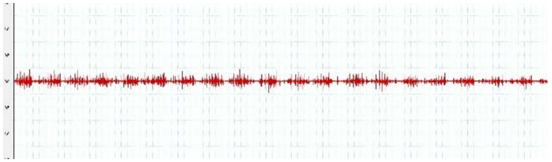

MEP dia

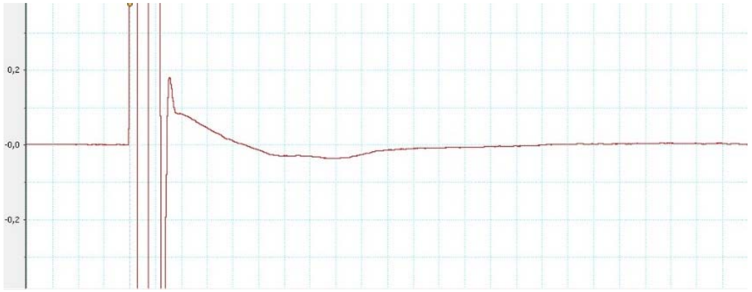

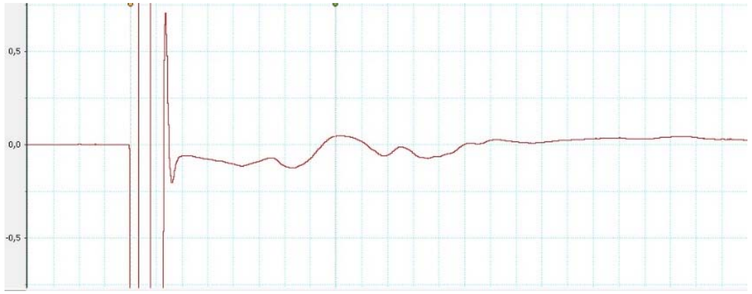

Sham animals

Rat #41

Ipsi

Contra

Diaphragm activity

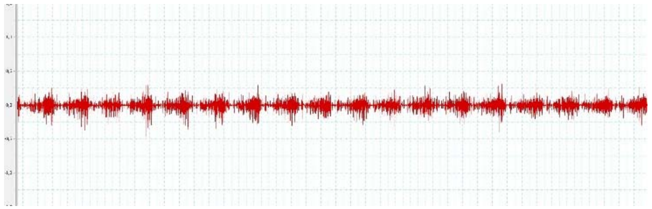

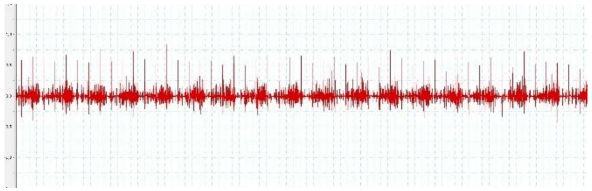

MEP dia

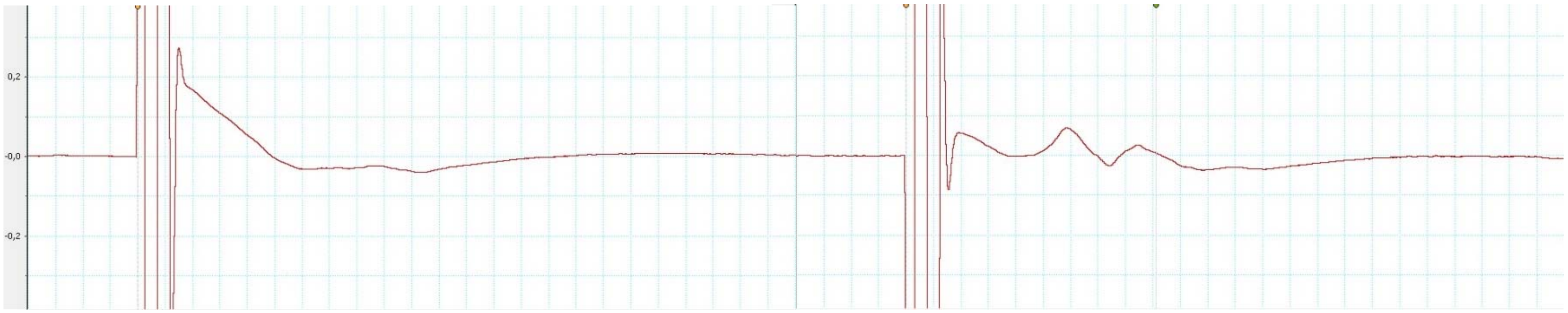

Sham animals

Rat #40

Ipsi

Contra

Diaphragm activity

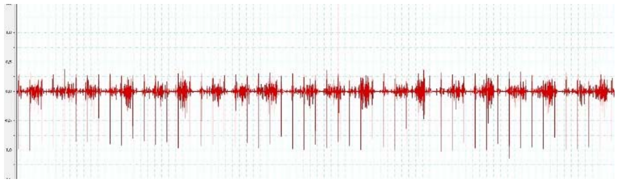

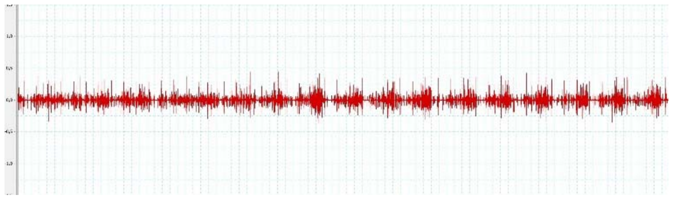

MEP dia

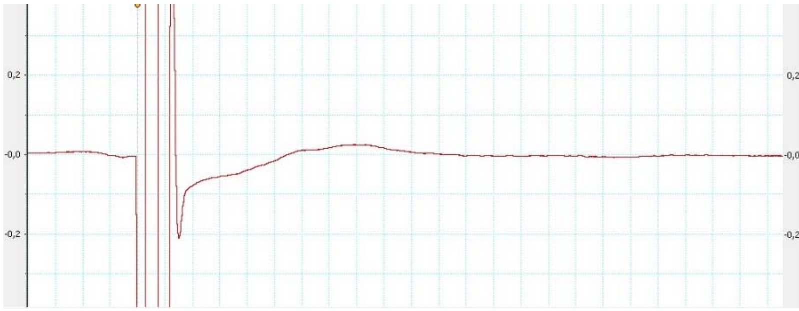

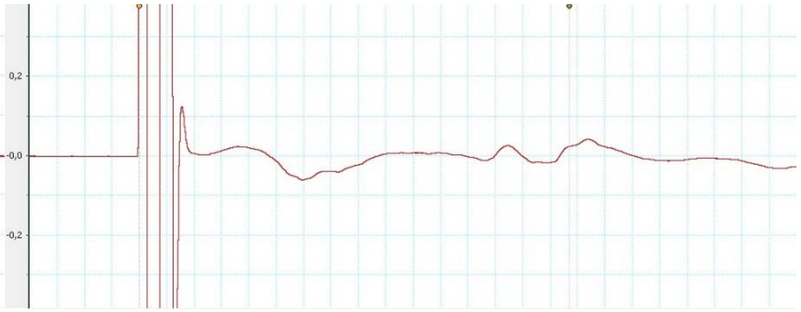

Sham animals

Rat #72

Ipsi

Contra

Diaphragm activity

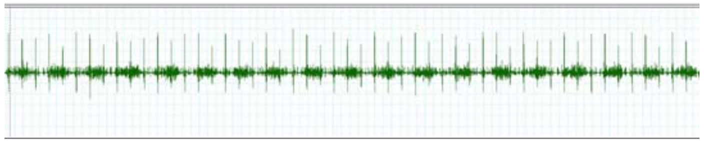

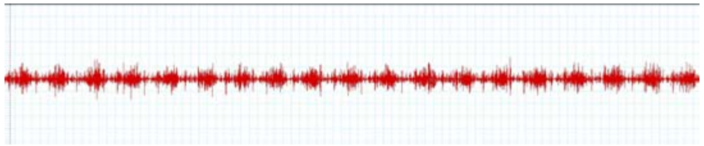

MEP dia

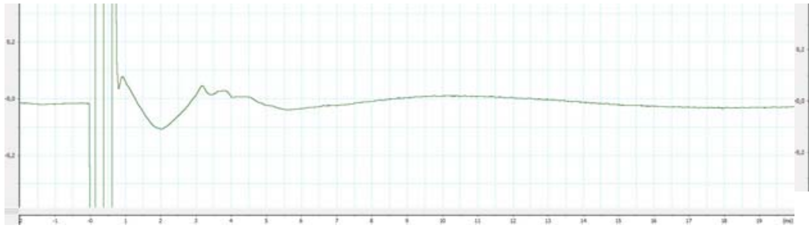

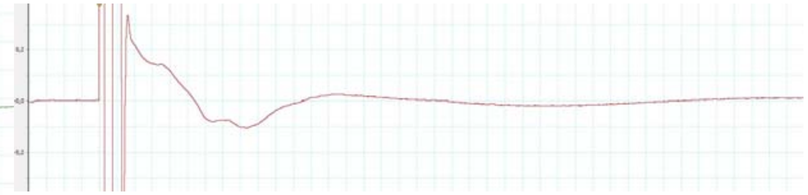

Sham animals

Rat #71

Ipsi | Contra

Diaphragm activity

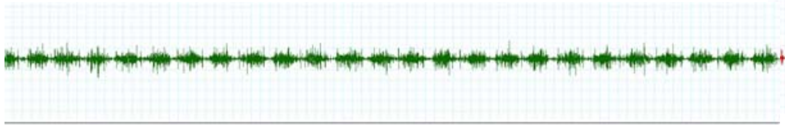

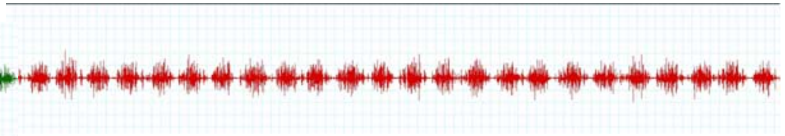

MEP dia

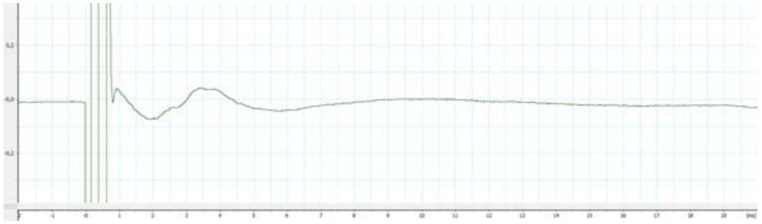

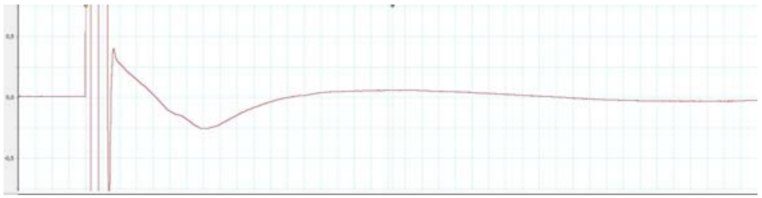

Sham animals

Rat #66

	Ipsi	Contra
Diaphragm activity	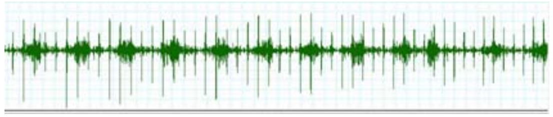	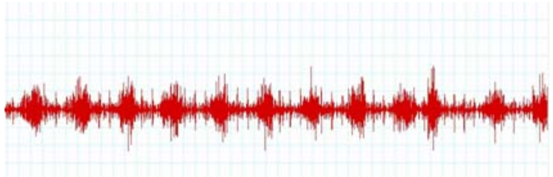
MEP dia	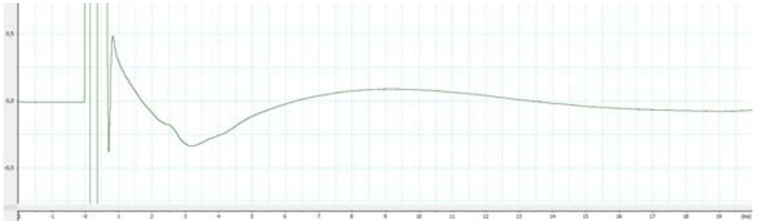	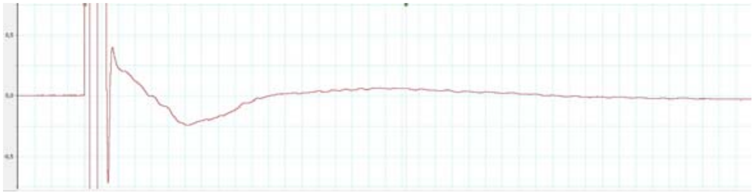

Sham animals

Rat #67

Ipsi

Contra

Diaphragm activity

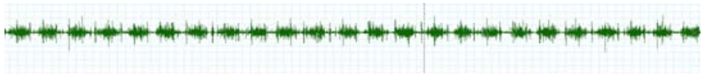

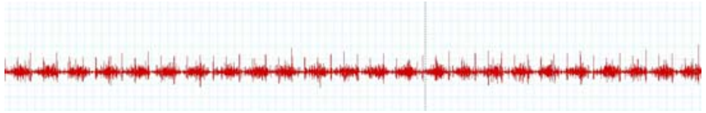

MEP dia

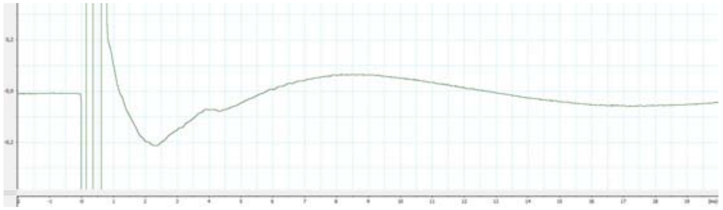

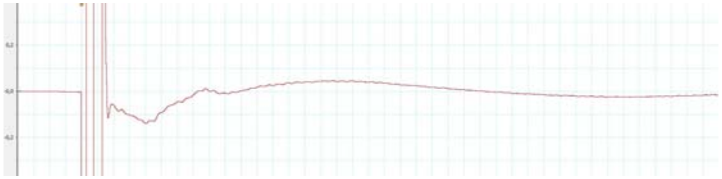

Sham animals

Rat #68

Ipsi

Contra

Diaphragm activity

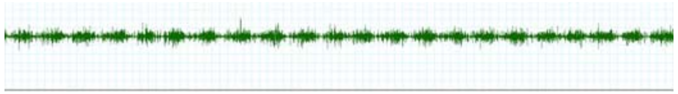

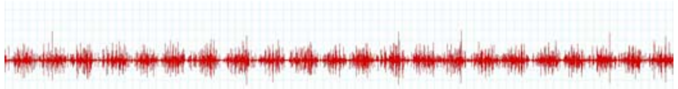

MEP dia

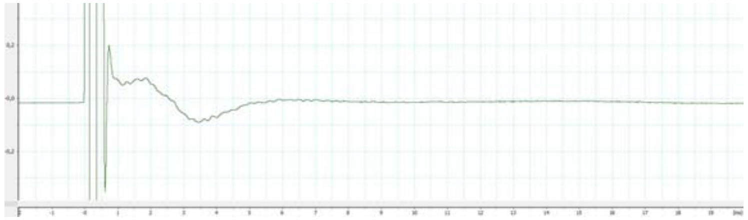

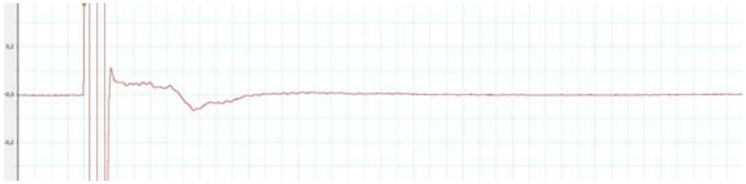

1h P.I.

Rat #27

Ipsi

Contra

Diaphragm activity

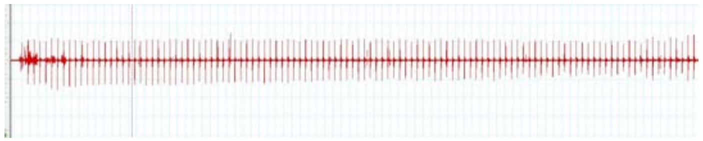

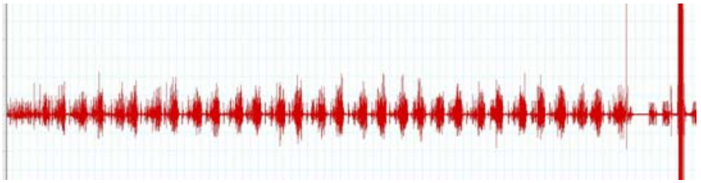

MEP dia

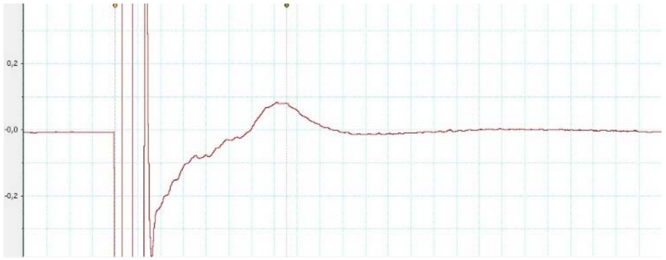

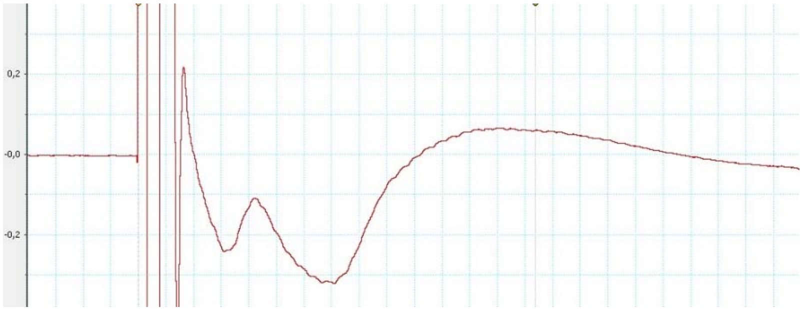

1h P.I.

Rat #29

	Ipsi	Contra
Diaphragm activity	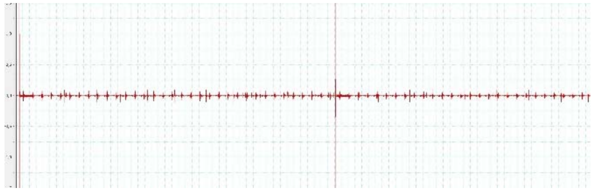	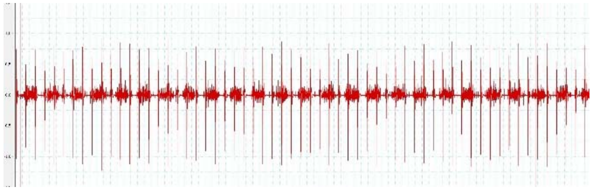
MEP dia	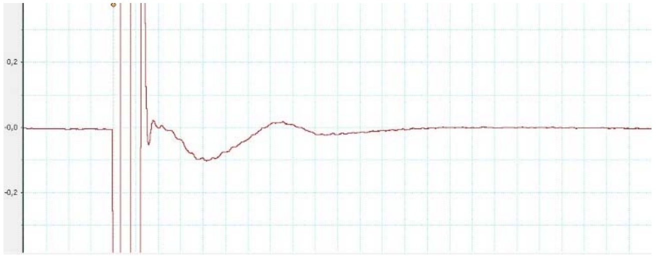	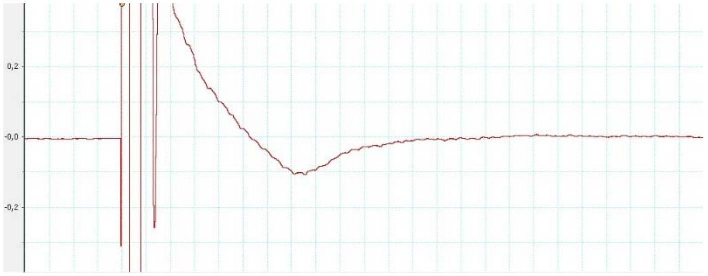

1h P.I.

Rat #30

	Ipsi	Contra
Diaphragm activity	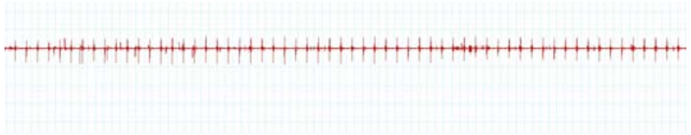	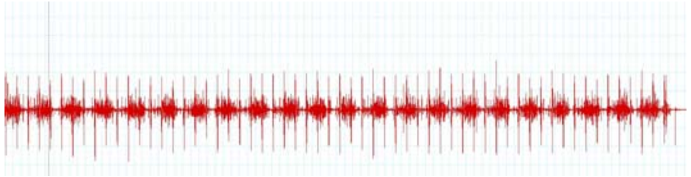
MEP dia	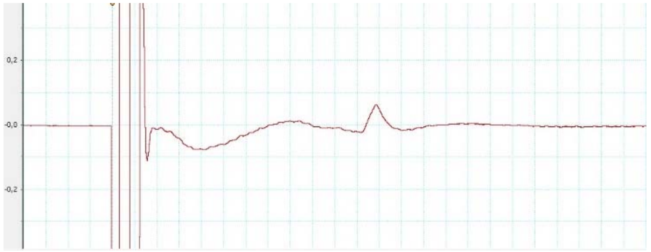	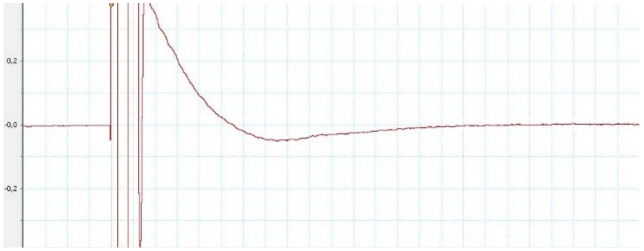

1h P.I.

Rat #31

Ipsi

Contra

Diaphragm activity

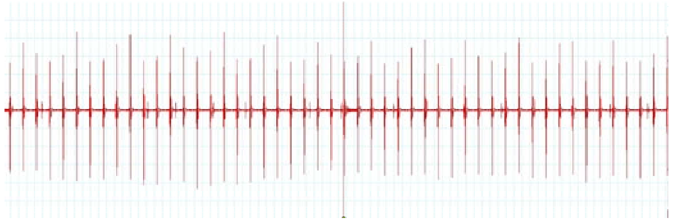

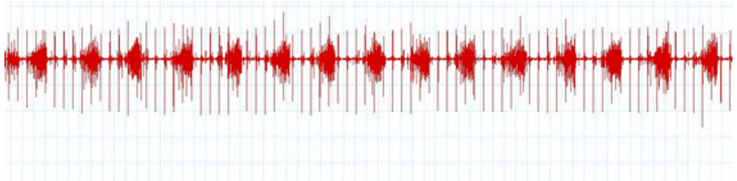

MEP dia

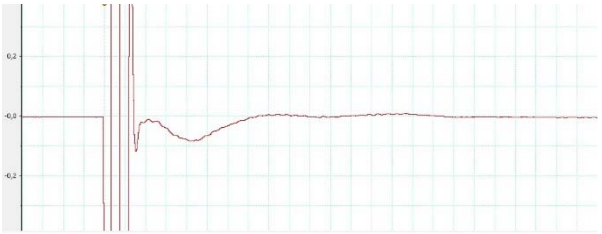

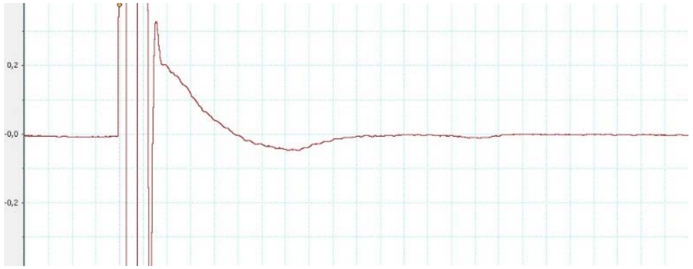

1h P.I.

Rat #32

Ipsi

Contra

Diaphragm activity

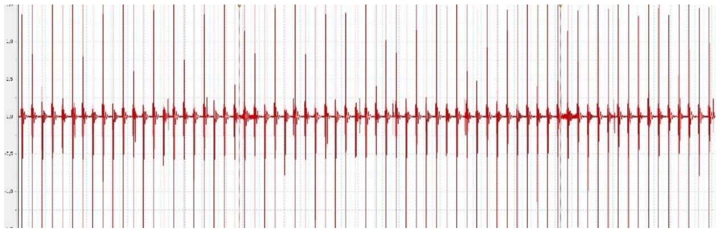

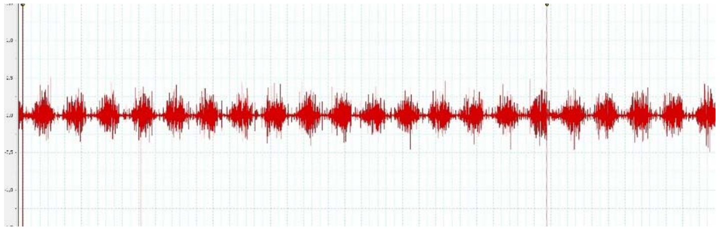

MEP dia

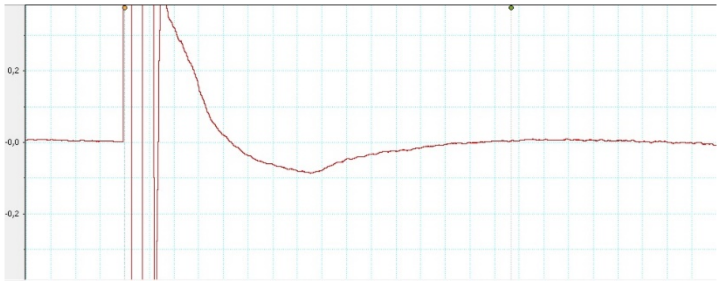

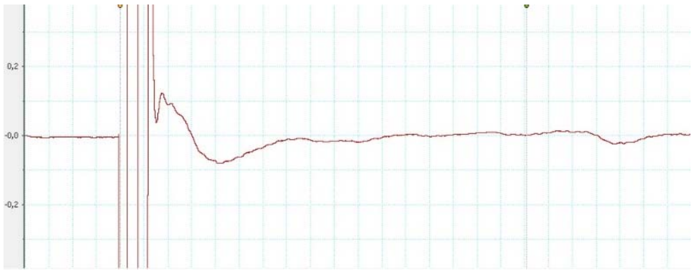

1h P.I.

Rat #33

Ipsi | Contra

Diaphragm activity

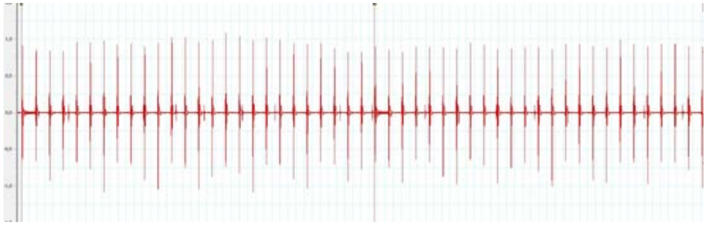

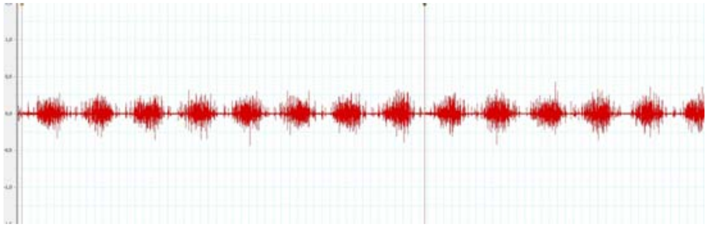

MEP dia

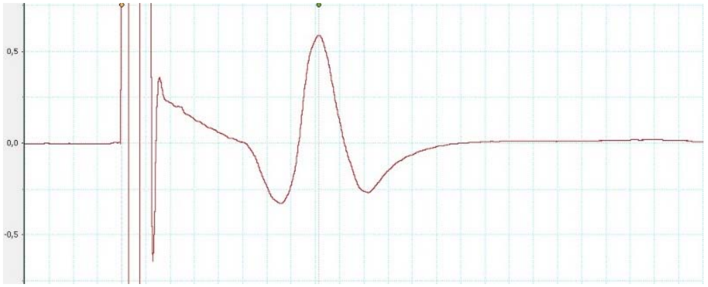

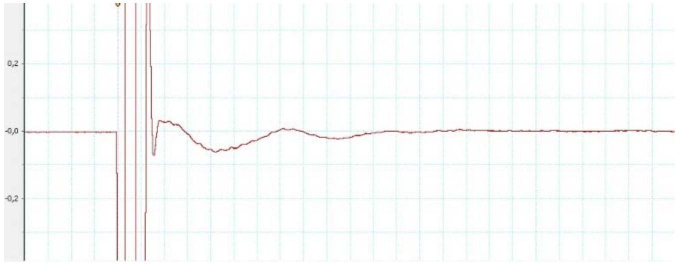

1h P.I.

Rat #69

	Ipsi	Contra
Diaphragm activity	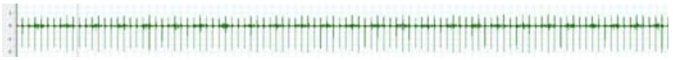	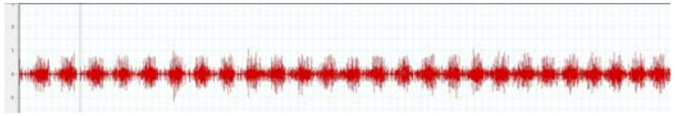
MEP dia	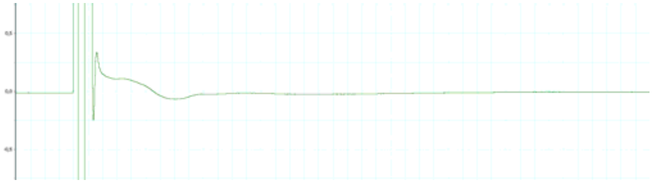	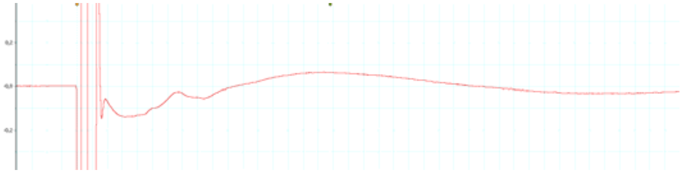

1h P.I.

Rat #70

Ipsi

Contra

Diaphragm activity

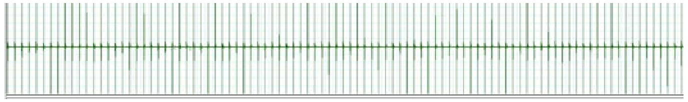

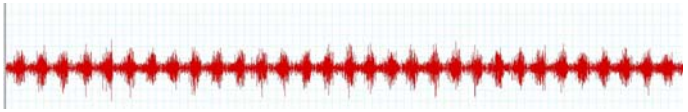

MEP dia

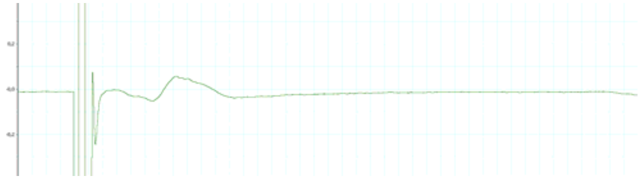

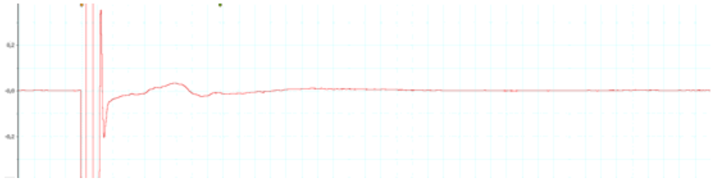

1h P.I.

Rat #73

	Ipsi	Contra
Diaphragm activity	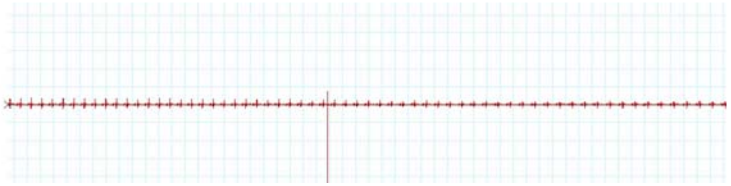	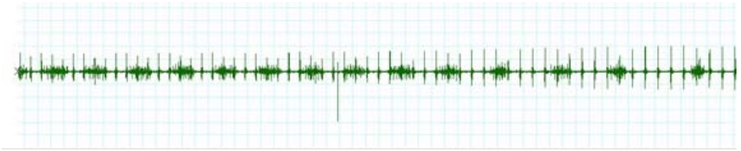
MEP dia	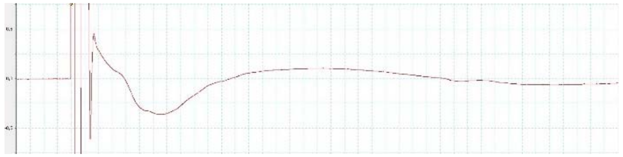	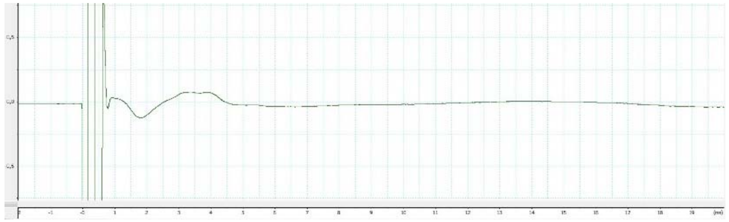

1h P.I.

Rat #74

	Ipsi	Contra
Diaphragm activity	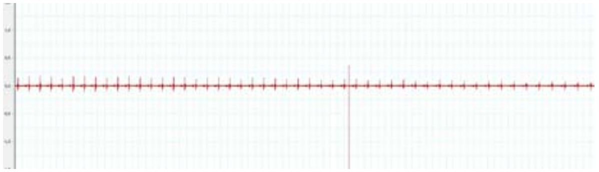	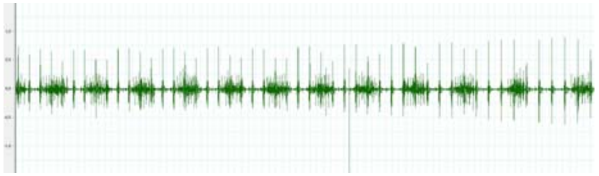
MEP dia	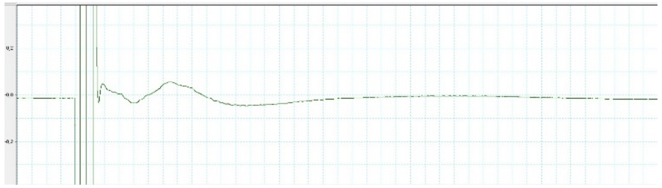	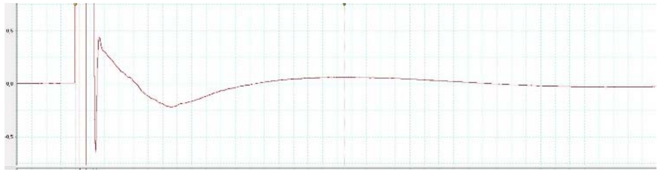

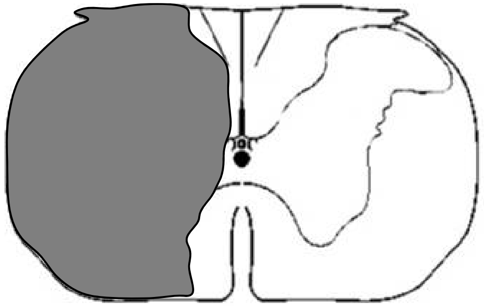

7 d P.I.

Rat #37

Ipsi

Contra

Diaphragm activity

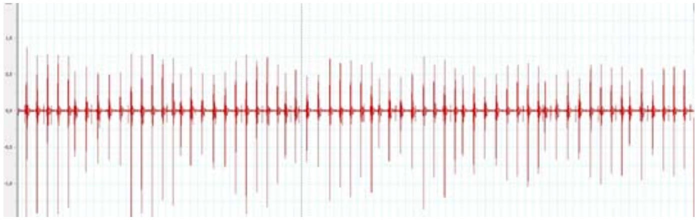

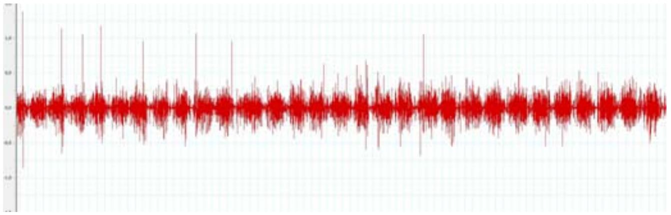

MEP dia

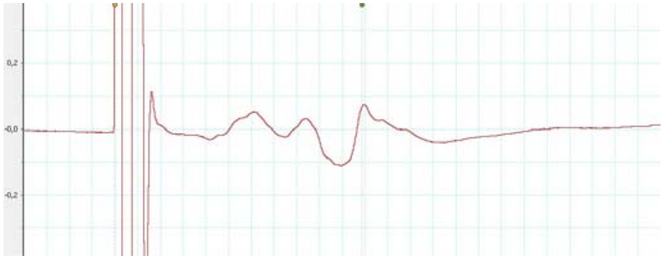

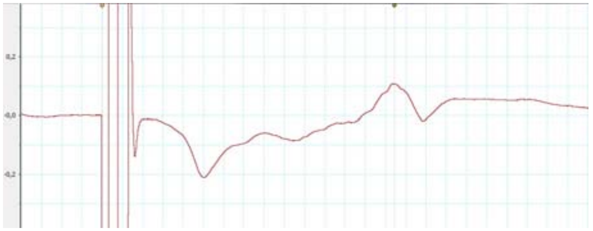

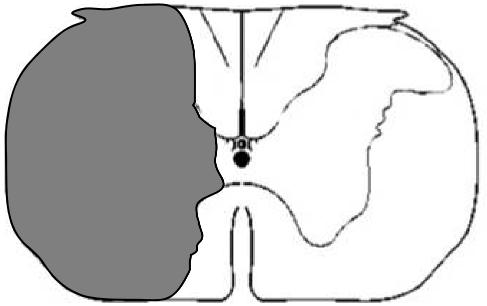

7 d P.I.

Rat #38

	Ipsi	Contra
Diaphragm activity	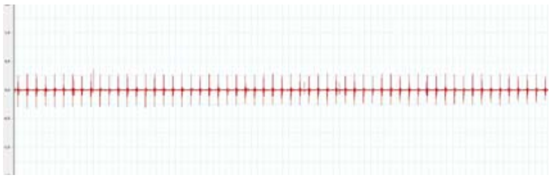	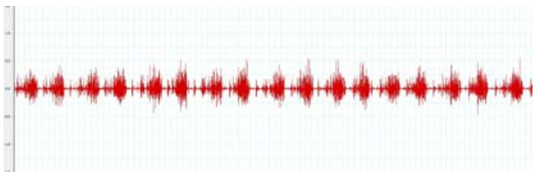
MEP dia	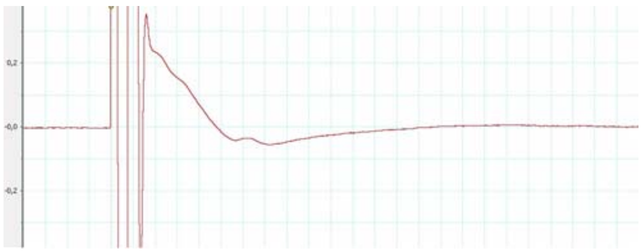	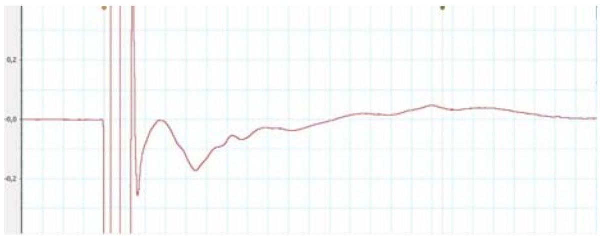

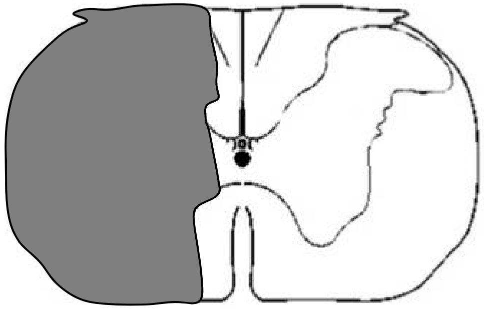

7 d P.I.

Rat #39

	Ipsi	Contra
Diaphragm activity	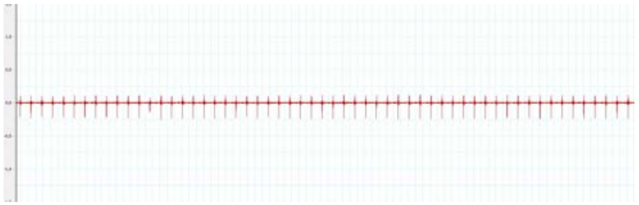	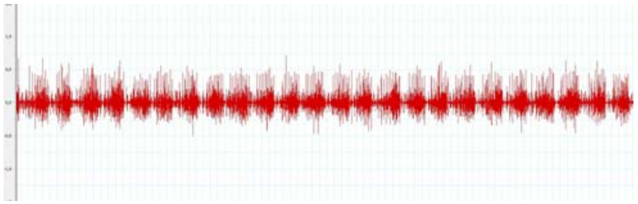
MEP dia	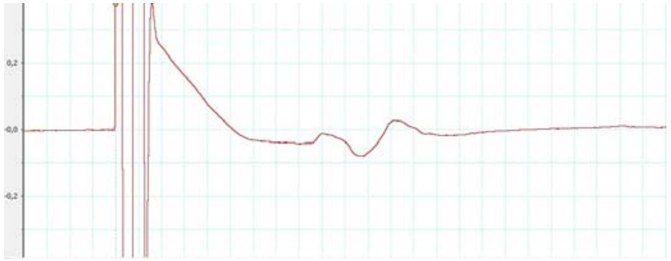	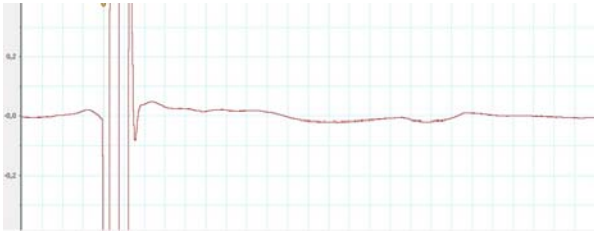

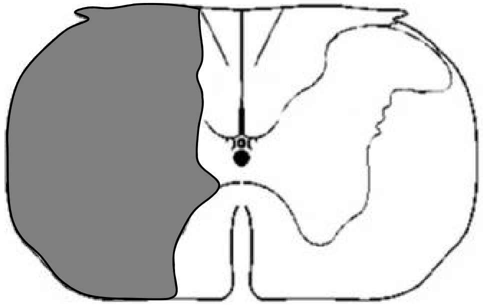

7 d P.I.

Rat #43

Ipsi

Contra

Diaphragm activity

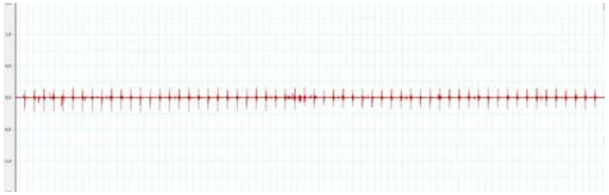

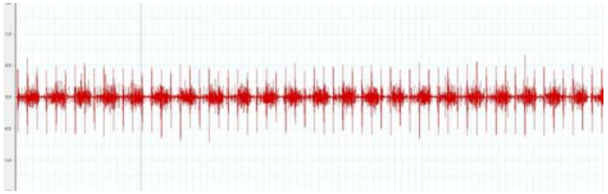

MEP dia

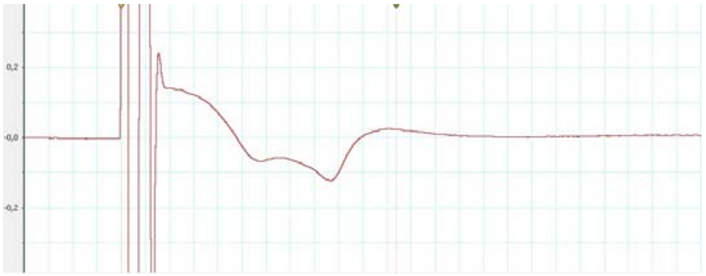

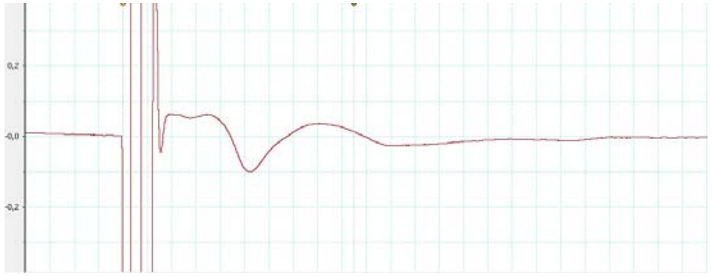

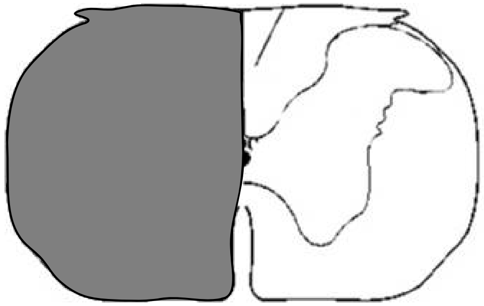

7 d P.I.

Rat #46

Ipsi

Contra

Diaphragm activity

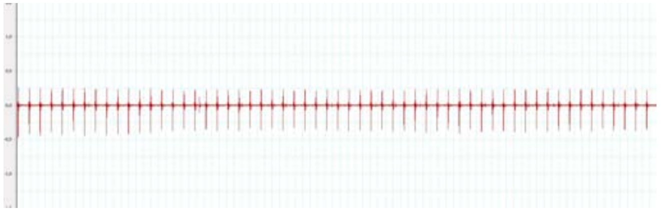

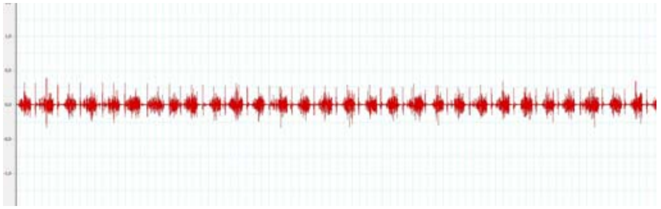

MEP dia

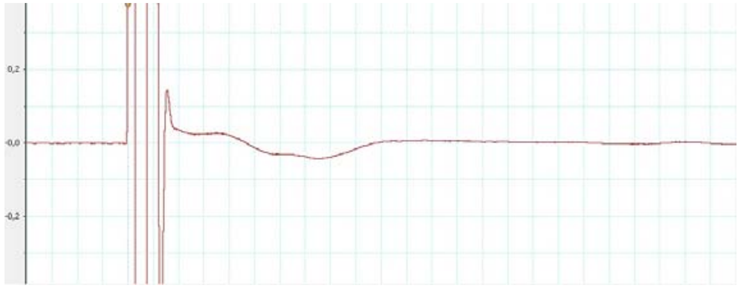

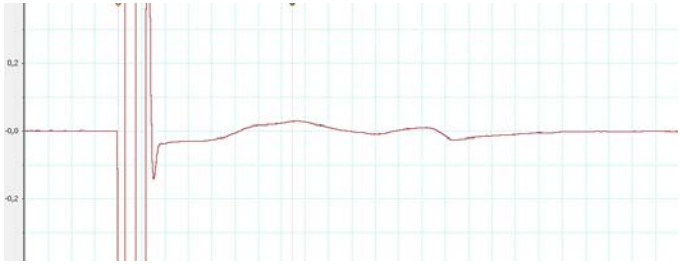

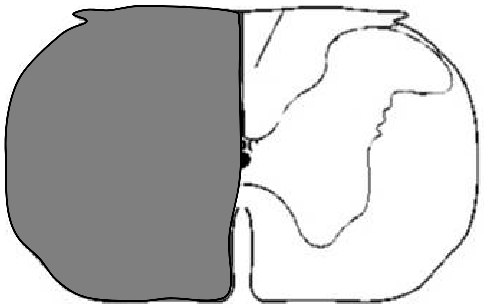

7 d P.I.

Rat #47

Ipsi

Contra

Diaphragm activity

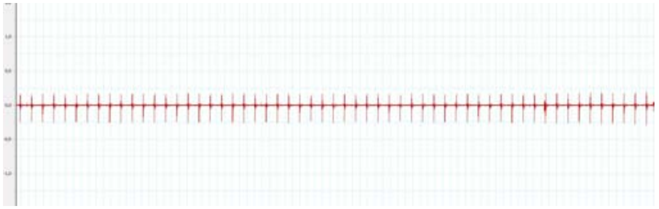

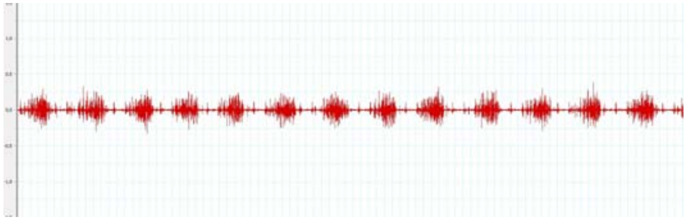

MEP dia

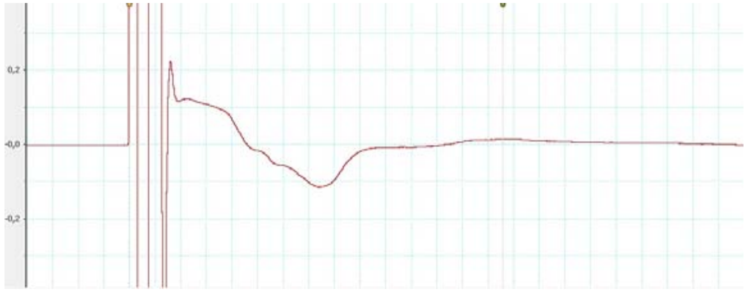

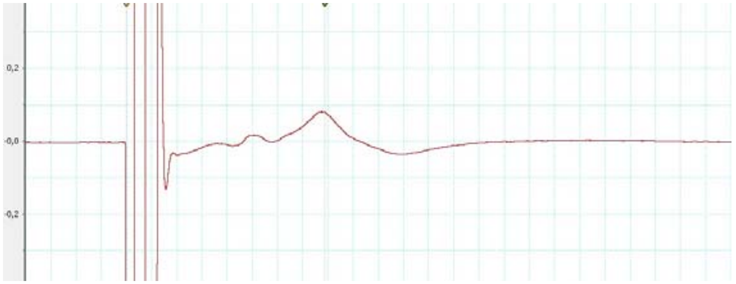

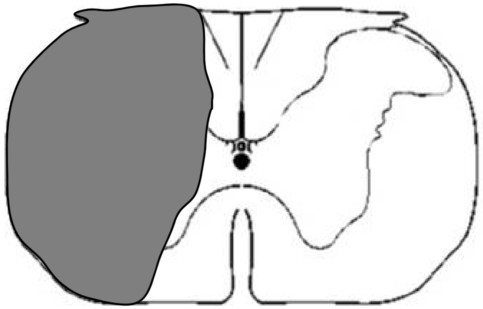

7 d P.I.

Rat #50

Ipsi

Contra

Diaphragm activity

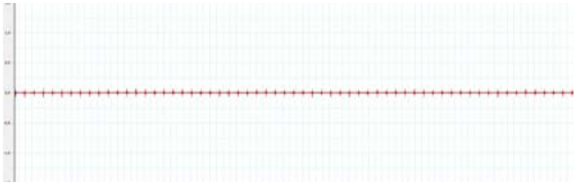

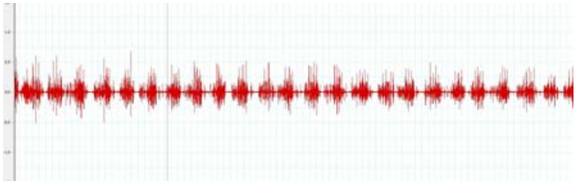

MEP dia

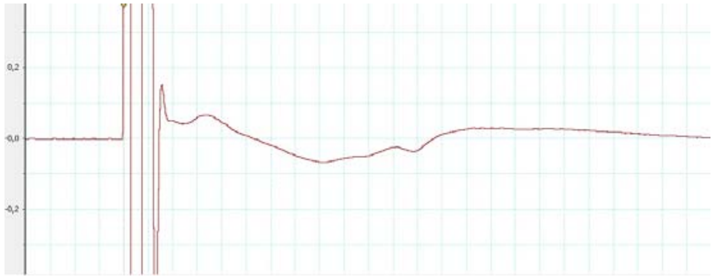

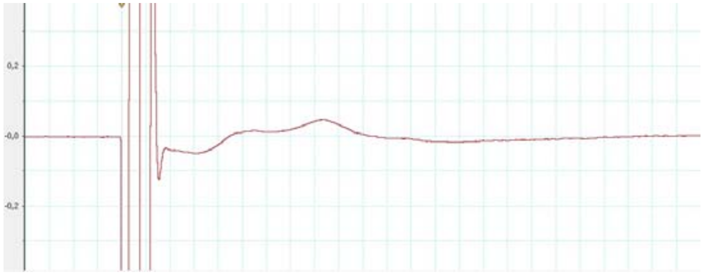

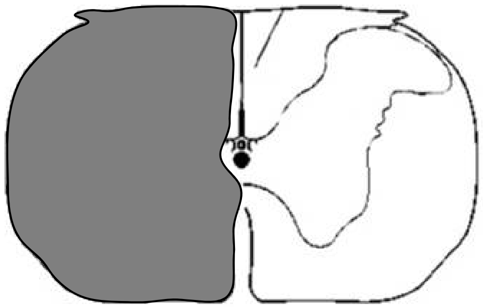

7 d P.I.

Rat #51

Ipsi

Contra

Diaphragm activity

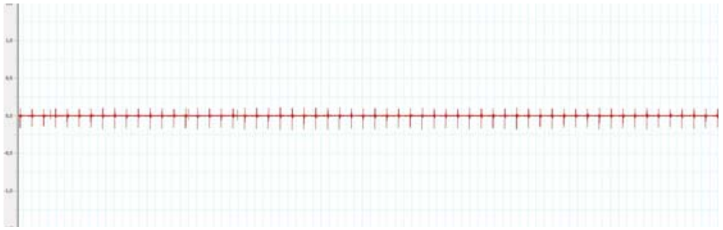

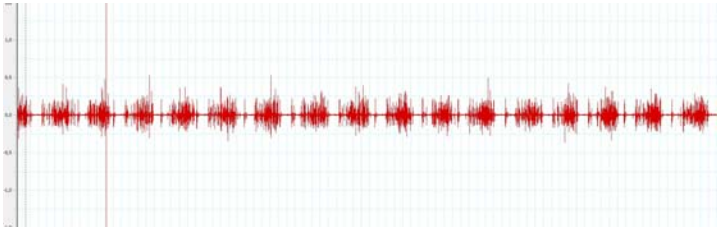

MEP dia

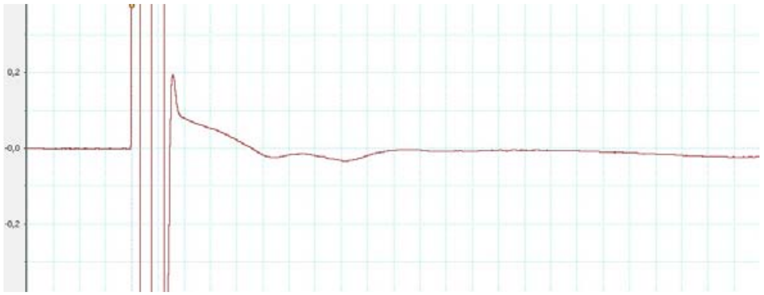

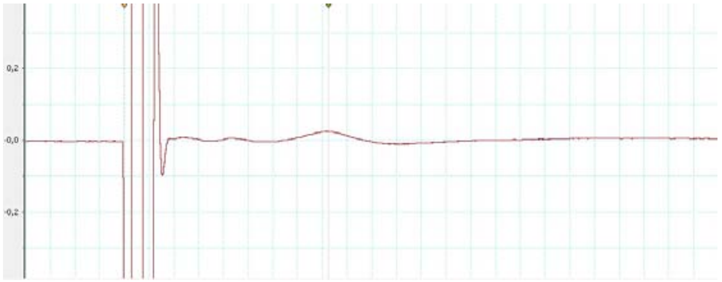

Supplement: S1 Supporting Information — (PDF) [file pone.0148180.s001.pdf]
